# A Randomised Trial Evaluating the Effects of the TRPV1 Antagonist SB705498 on Pruritus Induced by Histamine, and Cowhage Challenge in Healthy Volunteers

**DOI:** 10.1371/journal.pone.0100610

**Published:** 2014-07-21

**Authors:** Rachel A. Gibson, Jon Robertson, Harshna Mistry, Stewart McCallum, Disala Fernando, Melody Wyres, Gil Yosipovitch

**Affiliations:** 1 Academic Discovery Performance Unit, GlaxoSmithKline, Stevenage, Hertfordshire, United Kingdom; 2 Quantitative Sciences, GlaxoSmithKline, Stevenage, Hertfordshire, United Kingdom; 3 Clinical Pharmacology Science and Study Operations GlaxoSmithKline, Cambridge, Cambridgeshire, United Kingdom; 4 Academic Discovery Performance Unit, GlaxoSmithKline, Collegeville, Pennsylvania, United States of America; 5 Clinical Unit, GlaxoSmithKline, Cambridge, Cambridgeshire, United Kingdom; 6 Clinical Development, Stiefel, a GSK company, Research Triangle Park, North Carolina, United States of America; 7 Department of Dermatology and Temple Itch Center, Temple University School of Medicine, Philadelphia, Pennsylvania, United States of America; Gentofte University Hospital, Denmark

## Abstract

**Background:**

Transient receptor potential vanilloid type 1 (TRPV1) is a non-selective cation channel widely expressed in skin tissues, and peripheral sensory nerve fibres. Activation of TRPV1 releases neuropeptides; the resulting neurogenic inflammation is believed to contribute to the development of pruritus. A TRPV1 antagonist has the potential to perform as an anti-pruritic agent. SB705498 is a TRPV1 antagonist that has demonstrated *in vitro* activity against cloned TRPV1 human receptors and when orally administered has demonstrated pharmacodynamic activity in animal models and clinical studies.

**Objectives:**

To select a topical dose of SB705498 using the TRPV1 agonist capsaicin; to confirm engagement of the TRPV1 antagonistic action of SB705498 and assess whether the dose selected has an effect on itch induced by two challenge agents.

**Methods:**

A clinical study was conducted in 16 healthy volunteers to assess the effects of 3 doses of SB705498 on skin flare induced by capsaicin. Subjects with a robust capsaicin response were chosen to determine if the selected topical formulation of SB705498 had an effect on challenge agent induced itch.

**Results:**

Following capsaicin challenge the greatest average reduction in area of flare was seen for the 3% formulation. This dose was selected for further investigation. Itch intensity induced by two challenge agents (cowhage and histamine) was assessed on the Computerised Visual Analogue Scale. The difference in average itch intensity (Weighted Mean Over 15 Mins) between the 3% dose of SB705498 and placebo for the cowhage challenge was −0.64, whilst the histamine challenge showed on average a −4.65 point change.

**Conclusions:**

The 3% topical formulation of SB705498 cream was clinically well tolerated and had target specific pharmacodynamic activity. However there were no clinically significant differences on pruritus induced by either challenge agent in comparison to placebo. SB705498 is unlikely to be of symptomatic benefit for histaminergic or non-histaminergic induced itch.

**Trial Registration:**

ClinicalTrials.gov NCT01673529

## Introduction

Pruritus (itching) is a common symptom of skin disease and can best be defined as an unpleasant cutaneous sensation that leads to a desire to scratch [1], [2]. It can also be a common symptom in systemic disease and psychiatric disorders. All human beings experience pruritus in the course of their lifetime.

Chronic itch, which lasts for longer than 6 weeks, has a profound impact on quality of life, including detrimental effects on sleep, attention, and sexual function. At present, there is no universally accepted effective therapy for itch.

Historically, the neuronal pathways for itch have been principally characterised by responses to histamine. Intracutaneous application of histamine produces intense itch and a large area of axon-reflexive vasodilation (“flare”) around the application site. Both phenomena are thought to be mediated through neuronal activity in itch-specific, mechanoinsensitive C-fibre afferents(CMi). However, mechanical and electrical stimuli that do not activate CMi fibres can cause the sensation of itch, and itch may occur without flare, suggesting that other neuronal itch pathways exist [3]. There are many direct mediators of itch and there may be redundant systems. Numerous publications have identified the Transient Receptor Potential (TRP) channels (e.g. TRPV1, TRPV3, TRPA1, TRPM8) as having a key role in pruritus (for review see [4]–[8]) and Atopic Dermatitis (AD). TRPV1 has been shown to be up-regulated in AD-skin lesions, and the activation of TRPV1 causes the release of proinflammatory and pruritic mediators [7], [9].Ultimately these channels are key in depolarizing itch sensing neurons independent of upstream (redundant) pathways. Blocking these channels has the potential to block the itch sensation

The TRPV1 receptor can be activated by the TRPV1 agonist capsaicin or endogenous inflammatory mediators. The TRPV1 receptor is expressed in skin tissue including keratinocytes and peripheral sensory nerve fibres (C and Aδ).

SB705498 is a selective potent TRPV1 antagonist [10] that has demonstrated in vitro antagonist activity against cloned human TRPV1 receptors and when orally administered has shown pharmacodynamic activity in animal models and in clinical studies of pain and nasal secretion. [11]–[14].

Two challenge agents (Histamine and Cowhage) were selected as they induce pruritus by different mechanisms and hence would allow exploration of the therapeutic potential of SB705498. Histamine is thought to initiate pruritus through activation of sensory neurons predominantly C-fibers and via activation of phospholipase A2 and 12-lipoxygenase [15] and is the key puritogen in urticarial skin diseases in which antihistamines are most effective [16]. However several skin disorders including atopic dermatitis are resistant to antihistamine therapies [17]; [18]. Cowhage spicules (*Mucuna pruriens*), act through a histamine independent pruriceptive neuronal pathway releasing a cysteine protease (mucunanin) that activates proteinase-activated receptor 2 (PAR2) and PAR4 [19] in nerve fibers and keratinocytes. PAR2 activation has been reported to modulate the expression and ion channel activity of TRPV1. [20]; [21]. Costa *et al*. [22] suggested trypsin injection could activate PAR-2 receptors, stimulating the release of several mast cell mediators which in turn may sensitise TRPV1 receptors on sensory nerves, transmitting itch sensations to the CNS. More recently Belghiti *et al*. [23] showed that activation of PAR2 signalling sensitizes nociceptors by augmenting the expression and activity of neuronal TRPV1 channels contributing to the persistence of a puritogenic state in a rat model of liver disease. PAR2 receptors and their ligands, serine proteases, have previously been demonstrated to have a significant role in the itch associated with AD [7]. The potential role for an anti-pruritic effect of a TrpV1 antagonist (PAC14028) has been assessed and found to have a positive impact on a PAR-2 mediated murine atopic dermatitis and itching models [24].

## Objectives

The study was designed as a two part study in order to address the role of TRPV1 in pruritus and investigate the therapeutic potential of SB705498.

Part A was designed to assess whether any of three doses (1%, 3% and 5%) of SB705498 were able to adequately reach the target activated by the TRPV1 agonist capsaicin and to evaluate the safety and tolerability of SB705498 compared to placebo. Only if Part A was positive was Part B of the study initiated, with the dose of SB705498 producing the largest and/or most consistent average reduction in flare in Part A. Part B was designed to assess the affect of SB705498 on itch intensity and duration of itch induced by challenge agents (cowhage and histamine) compared to placebo.

## Methods

The protocol for this trial and supporting CONSORT checklist are available as supporting information; See Checklist S1 and Protocol S1.

### Study Overview

The study was conducted as a single site, two part randomised, double-blind, placebo controlled trial. The study took place at GSK's Clinical Unit in Addenbrookes Hospital, Cambridge, United Kingdom, between July and October 2012. The clinical research was reviewed and approved by GSKs internal review panels and Independent Local Research Ethics Committee located in Brent, London, United Kingdom. Written informed consent was obtained prior to study recruitment and the investigations were conducted according to the principles expressed in the Declaration of Helsinki.

This study was open to adult (≥18 years old) male or female healthy volunteers. Screening involved review of medical history, physical examination and laboratory screening tests. The use of recreational drugs, alcohol and nicotine products, was restricted or prohibited. Participants were required to avoid UV exposure for 7 days prior to screening, during study conduct and for 7 days after the last dose. Subjects were not eligible for inclusion if they presented with any skin infection or inflammation on the forearm or suffered from any acute or chronic dermatological problems. In each treatment session subjects were screened for drugs of abuse and alcohol, vital signs, and ECG prior to all other assessments. All eligible participants were required to show a flare response to capsaicin and to have pruritus induced by both challenge agents (cowhage and histamine) prior to enrolment in the study. Details of the capsaicin and cowhage/histamine challenge are detailed under Screening.

The clinical study staff were blinded to the treatment until the study was completed, however specified members of the team had details of treatment allocation for safety purposes and also to allow for selection of subjects for further study (Double Blind (sponsor un-blind)).

### Sample Size

Part A was designed to randomise 16 subjects in a complete block 4-period crossover design. Sixteen subjects would ensure that the 95% confidence interval for the ratio between active and placebo for the area of flare as induced by capsaicin would be no wider than 37.7%, assuming a within subject coefficient of variation of 0.45.

Part B of the study was powered to detect a 20 point difference between the chosen dose of SB705498 and placebo on the 0–100 Computerized Visual Analogue Scale (COVAS, Medoc, Ramat-Yishai, Israel), assuming a within subject standard deviation of 11.5 with a two-sided type 1 error rate of 5%. Ten subjects were required for the crossover design.

### Randomisation Details

The centre based randomisation schedule for part A was created adopting a Williams design of a generalised Latin square, for a 4×4 crossover period design using a block size of 8. The four sequences were 1 2 4 3, 2 3 1 4, 3 4 2 1 or 4 1 3 2, where 1 was placebo and 2 to 4 were assigned to each increase in dose, and each treatment was assigned using an equal randomisation ratio.

The centre based randomisation schedule for part B, was an incomplete block crossover design using a block size of 8, as 8 sequences were created. An equal randomisation ratio was allocated between the sequences but given the number of subjects not all sequences were utilised twice. The sequences utilised were: E F E F, F E F E, E F F E or F E E F in a 1∶1∶1∶1 ratio where one of these were placebo and the other was SB705498.

### Allocation/Implementation

A randomisation sequence was generated by the GSK statistician and the randomisation schedule was sent to the clinical site. Based on treatment allocation detailed in the randomisation schedule, doses of the active cream or vehicle of the cream (placebo) was prepared by the pharmacy staff at the Clinical site.

Participants were enrolled into the study by recruitment staff at the site according to their standard procedure for healthy volunteer studies. Participants were identified via the site's healthy volunteer database. Once the participants passed screening they were assigned to the allocated interventions by the principal investigator.

The site staff and the participants were blinded to the treatment with the exception of the site pharmacy staff, who did the packaging and releasing of the cream or vehicle of the cream (placebo).

### Screening

At screening all eligible participants were required to show a flare response to capsaicin and to have pruritus induced by both challenge agents (cowhage and histamine) - scoring greater than 40 on the 0–100 COVAS. The COVAS allows rating of itch intensity on a 100 mm scale that ranges from ‘no itch’ at one end to ‘unbearable itch’ at the other [25]. All subjects were familiarised with study assessments, including any pharmaco-dynamic tests at the screening visit.

Thirty days were allowed between screening and Part A of the study and a maximum of 60 days between screening and Part B of the study. No subjects were required to re-screened prior to the start of Part B.

### Capsaicin Challenge

Approximately 0.5 mL of capsaicin cream (Axsain, 0.075% capsaicin w/w) was applied to a 3×3 cm square area on the volar aspect of one arm. The cream was left on the skin for 30 minutes and then gently wiped off. Assessments of skin blood flow were performed before and after capsaicin application by monitoring cutaneous blood flow using Laser Doppler imaging (LDI) (LDI-2, Moor Instruments Ltd., Devon, UK). A suitable area of approximately 16×8 cm around the stimulation site was scanned. The flare area (in cm^2^) was calculated from all pixels around the stimulation site in which flux values exceeded the 95% percentile (mean +2 SD) of the baseline distribution. The mean blood flow in the area of flare was also calculated using relative flux (arbitrary units).

### Cowhage

Approximately 70–100 cowhage spicules/fragments were counted for each application under a microscope to ensure between 30–35 spicules of sufficient quality for itch induction were available. These spicules were transferred from the microscope to folded paper troughs using disposable gel-loading tips and taken to the clinic on the paper trough in Petri dishes. The spicules were then transferred directly from the paper to a predefined 3x3 cm area on the volar aspect of the forearm by holding the paper vertically and tapping gently with a suitable implement e.g. a pen (being careful not to inadvertently flick the paper and disperse spicules).

The spicules were gently rubbed with a gloved finger for 45 seconds onto the subject's skin with a circular motion to facilitate contact. Approximately 1–2 minutes before contact with the cowhage spicules subjects were asked to start rating their itch intensity using the 0–100 COVAS. The itch intensity was recorded for 15 minutes or until an itch score of 0 (baseline) was recorded for 60 continuous seconds. Recording of pruritus did not exceed 15 minutes.

### Histamine

A 1% solution of histamine was applied using the skin prick method. [26]. This is a widely accepted method used in allergy clinics to test for histamine sensitivity; causing pruritus, skin flare and a skin wheal. The formulation and dose of histamine was based on methodology used in similar studies of itch [25]. The skin prick method involves placing a drop of 1% histamine solution onto the skin and using a lancet to gently pierce the superficial layer of the skin. The excess histamine solution was then wiped away. Itch intensity was rated using the COVAS as previously described.

### Design of Part A

16 subjects were recruited in Part A and were required to participate in a capsaicin challenge.

Subjects received individual applications of one of three doses of 1%, 3% and 5% of SB705498 as well as a placebo in a randomised order on four discrete 3×3 cm square patches on the volar surface of both forearms over 2 days (Two applications were applied each day). The topical application of SB705498 or placebo was left on the arm for 1 hour. Following the procedures described under screening the capsaicin was applied and assessments of skin blood flow (flare) were performed using LDI. A baseline LDI scan was performed prior to the application of any cream (SB705498 or Placebo) and again after 1 hr once the excess cream had been wiped away. A third scan occurred 5 minutes after the capsaicin challenge (once excess capsaicin has been wiped away). A final scan took place 2 hours post application of the SB705498/Placebo. A reduction in flare compared to placebo was considered a positive study outcome for Part A.

The decision to progress to Part B of the study was made by the unblinded members of the study team after reviewing the data from Part A. A reduction in area of flare for subjects receiving SB705498 compared with placebo was required to be observed on at least one of the dose strengths studied. The dose level deemed to have the largest and most consistent effect was the 3% cream and this was the dose strength studied in Part B.

### Design of Part B

Of the sixteen subjects randomised to participate in Part A, ten subjects who passed a second round of screening and who showed the most optimal treatment response during the capsaicin challenge (Part A) compared to placebo were asked to participate in Part B. Each volunteer was randomised to receive the placebo and the 3% SB705498 cream for both the cowhage and histamine challenge. Either the placebo or active cream was applied 1 hour before treatment with the challenge agent. Between itch inductions a break was taken to allow previous itch sensations to completely subside.

Each subject participated in both a cowhage and a histamine challenge. Challenge agents were applied on consecutive days with both treatment arms in a crossover fashion. Full details of the application process for the cowhage and histamine are described under screening.

### Statistical Analysis

#### Part A

The primary endpoint, area of flare, was log transformed prior to analysis and analysed via a mixed effects model, fitting treatment and period as fixed effects and subject as a random effect, calculating pair wise comparison for each active dose against placebo. Ratios to placebo and 95% confidence intervals of the ratio of the difference between each active dose strength and placebo were calculated.

#### Part B

The primary endpoint of average itch was calculated over the 15 minute assessment period, as a weighted mean, weighting the itch scores depending on the amount of time between each itch intensity assessments. This was analysed using a mixed effects model, fitting terms for treatment and period as fixed effects and subject as a random effect. A separate model was performed for each stimulant. No baseline was fitted in the model since no itch was present until the stimulant was added. Point estimates and 95% percent confidence intervals for the difference between and placebo were constructed.

Upon inspection of the data it was decided to perform an additional analysis of the average itch over the itch period only, as determined by the time of itch onset until the time to zero itch or the end of the challenge period. This was due to less itch than expected being reported over the 15 minute assessment period.

Descriptive statistics were used to describe all endpoints in Part B, including time to onset, time for itch to return to zero, duration of itch, time to peak itch, peak itch intensity and duration of peak itch.

## Results

### Enrolment and baseline Characteristics

Between July and October 2012 a total of 45 healthy volunteers (≥18 years old) were screened and 16 white male subjects were recruited into Part A of the study. Whilst male and female subjects were eligible to participate only male subjects were recruited into this study. Only volunteers that were sensitive to capsaicin i.e. develop flare on application of 0.5 ml of Axsain and had a score of ≥40 on the COVAS for both histamine and cowhage were enrolled.

**Figure 1:**
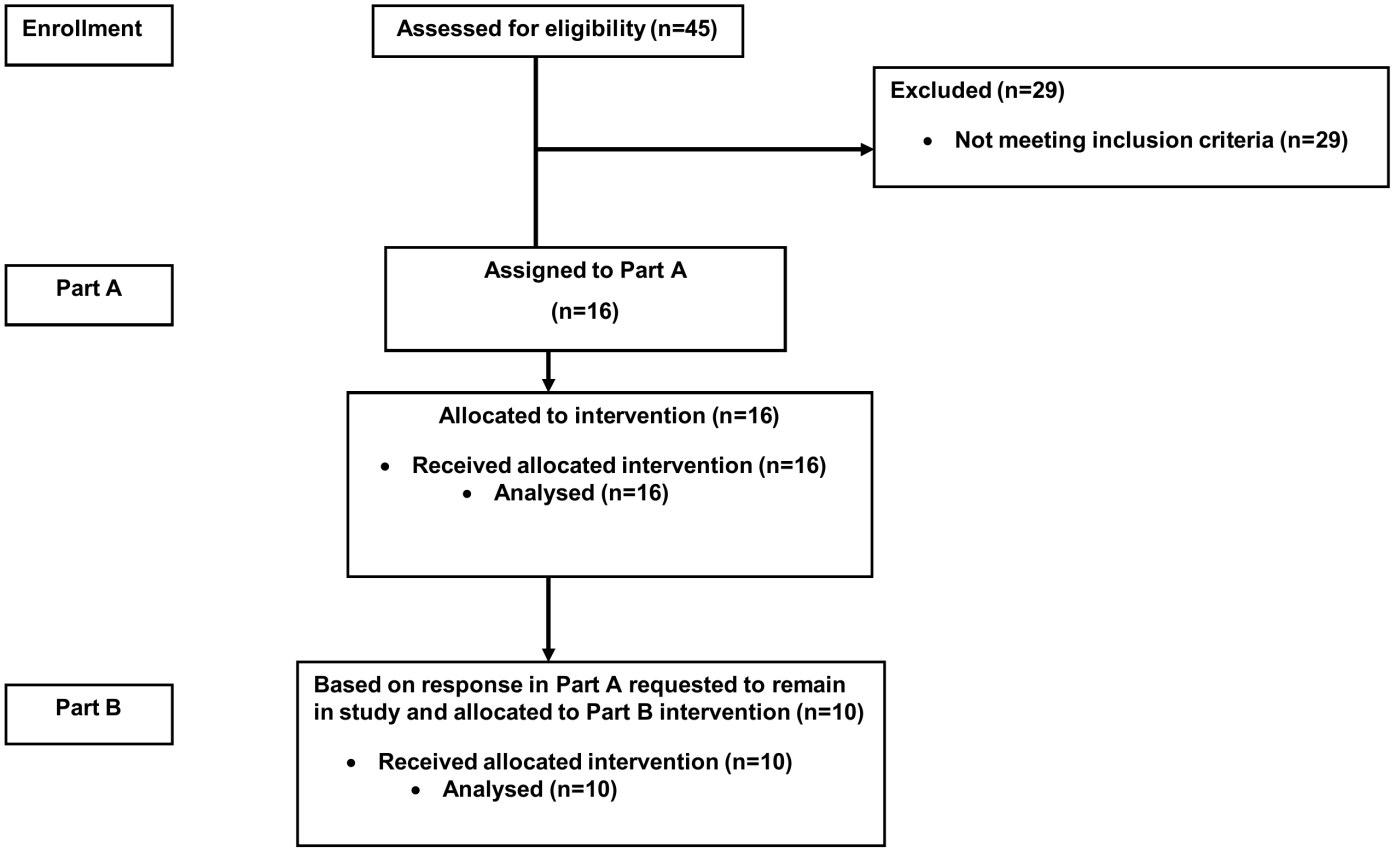
CONSORT diagram.

Ten of the best available responders were identified and subsequently invited to participate in Part B (See Figure 1). Demographic data for the cohort is provided in Table 1.

**Table 1:** Demographics and Baseline Characteristics.

	Part A	Part B
**Demographics**		
Age in Years, Mean (Range)	39.4 (30,52)	39.9 (30,52)
**Sex, n**		
Female:	0	0
Male:	16	10
**BMI** (kg/m^2^), Mean (Range)	26.36 (22.9,30.7)	25.97 (23.3,30.7)
**Height** (cm), Mean (Range)	177.6 (162,189)	178.7 (162,189)
**Weight** (kg), Mean (Range)	83.1 (69,100)	82.8 (71,100)
**Ethnicity, n (%)**		
Hispanic or Latino:	0	0
Not Hispanic or Latino:	16 (100%)	10 (100%)
**Race, n (%)**		
White – White/Caucasian/European Heritage	16 (100%)	10 (100%)

### Pharmacodynamic Results

Plasma/serum analysis revealed no quantifiable drug above the detection limit of the assay (0.5 ng/mL) and as such no PK analysis could be conducted. No clinically significant drug related AE's or any SAE's were reported from either part of the study.

### Part A

#### Area of Flare

**Figure 2:**
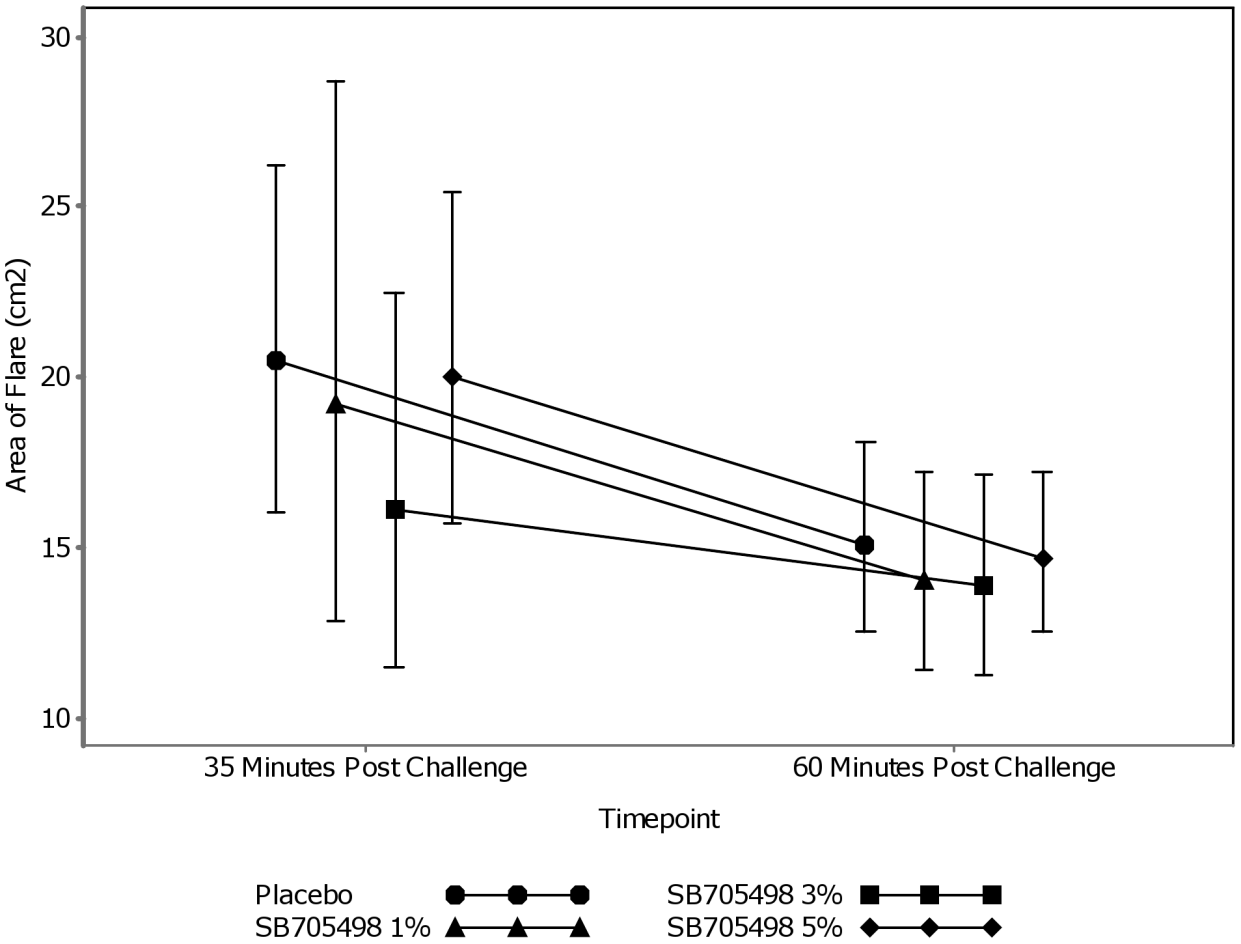
Geometric Mean Profile Plot of Area of Flare.

No clear dose response was observed at either of the post challenge timepoints for the three doses of SB705498 and placebo and therefore conclusions were drawn from the mixed effects model. Figure 2 shows the geometric mean profile with 95% C.I.of area of flare for placebo and the 1%, 3% and 5% doses of SB705498.

Adjusted geometric means, produced from the statistical analysis, for each treatment arm and for both timepoints are shown in Table 2. For the 35 minute post capsaicin challenge timepoint the biggest difference observed between the active doses and the placebo was seen on the 3% cream. Table 3 shows the adjusted ratio of the treatment difference was 0.78 (95% C.I. (0.52, 1.18) indicating a 22% average reduction in area of flare for 3% compared with placebo. Based on the data, the probability there was any treatment effect for this dose strength (ratio<1) was 88%. The 1% and 5% cream showed a small beneficial effect when compared with the placebo cream.

**Table 2:** Adjusted Geometric Means from Statistical Analysis of Area of flare.

Challenge	Treatment	N	n	Adjusted means (SE Logs)	95% Confidence Interval
35 Mins Post Challenge	Placebo	16	16	20.49 (0.148)	(15.24, 27.56)
	SB705498 1%	16	16	19.18 (0.148)	(14.26, 25.80)
	SB705498 3%	16	16	16.07 (0.148)	(11.95, 21.61)
	SB705498 5%	16	16	19.98 (0.148)	(14.86, 26.87)
60 Mins Post Challenge	Placebo	16	16	15.06 (0.089)	(12.60, 17.99)
	SB705498 1%	16	16	14.04 (0.089)	(11.75, 16.78)
	SB705498 3%	16	16	13.90 (0.089)	(11.63, 16.62)
	SB705498 5%	16	16	14.70 (0.089)	(12.30, 17.57)

**Table 3:** Treatment Comparisons from Statistical Analysis of Area of Flare.

Challenge	Comparison	Adjusted Ratio(SE Logs)	95% Confidence Interval	Probability Ratio^1^		
				<1	<0.9	<0.7
35 Mins Post Challenge	SB705498 1% - Placebo	0.94 (0.205)	(0.62, 1.41)	0.63	0.42	0.22
	SB705498 3% - Placebo	0.78 (0.205)	(0.52, 1.18)	0.88	0.75	0.54
	SB705498 5% - Placebo	0.98 (0.205)	(0.65, 1.47)	0.55	0.35	0.17
60 Mins Post Challenge	SB705498 1% - Placebo	0.93 (0.119)	(0.73, 1.19)	0.72	0.38	0.10
	SB705498 3% - Placebo	0.92 (0.119)	(0.73, 1.17)	0.75	0.41	0.12
	SB705498 5% - Placebo	0.98 (0.119)	(0.77, 1.24)	0.58	0.25	0.05

1 Posterior Probability the treatment ratio is less than the stated number. This was calculated assuming non-informative priors.

For the 60 minute post capsaicin challenge timepoint the beneficial treatment effect for the 3% cream reduced to a comparable level with the 1% and 5% cream. All 3 doses showed minor benefit when compared to the placebo.

Given these results it was decided to progress the 3% cream through to Part B of the study.

### Part B

#### Itch Intensity (Weighted Mean over 15 Mins)

**Figure 3:**
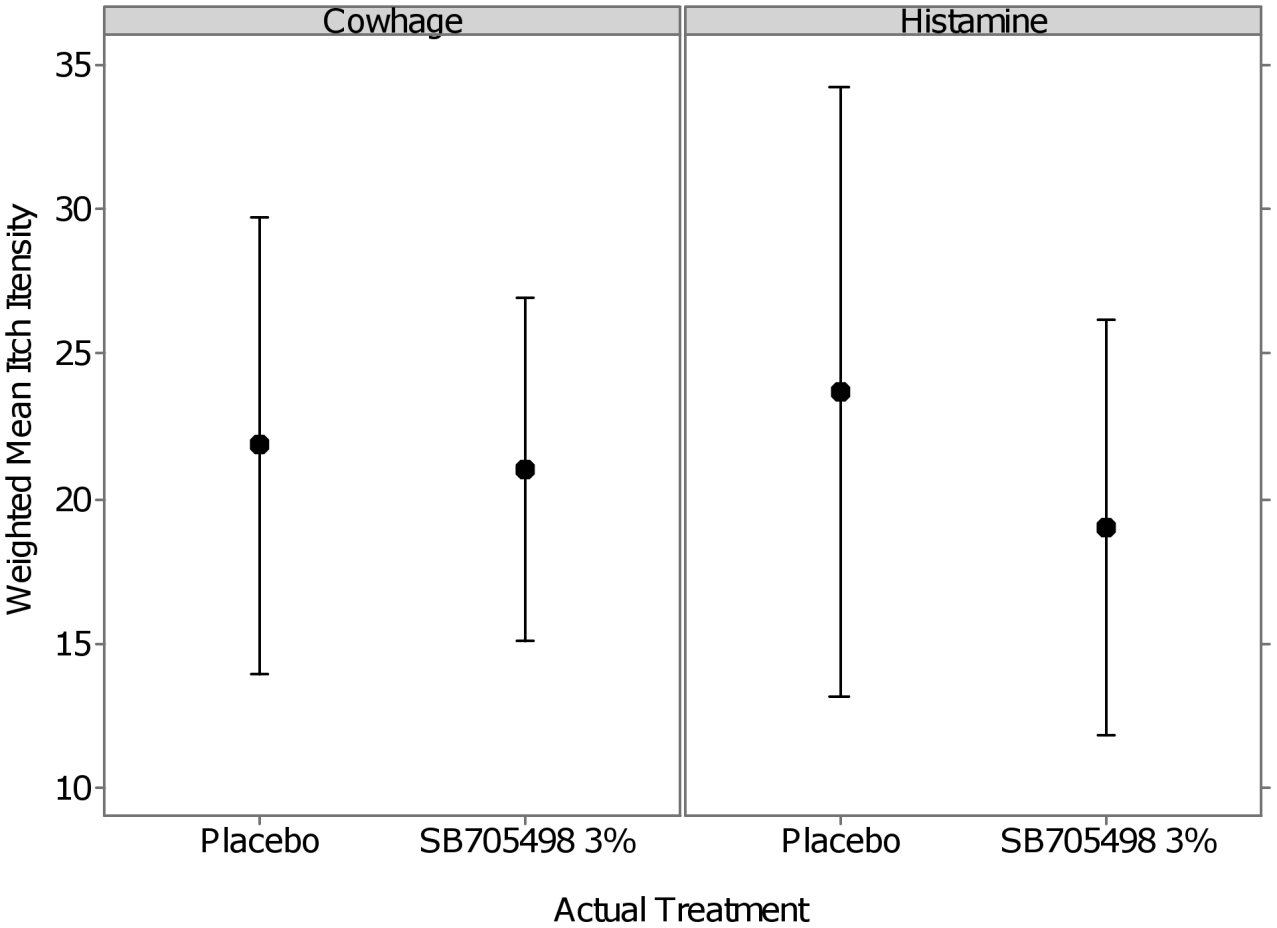
Mean profile plot of average itch intensity (weighted mean over 15 minutes).

The mean average itch intensity as defined as the weighted itch scores over the 15 min assessment window (95% C.I.) to both the cowhage and histamine challenge can be seen in Figure 3. The mean responses for the cowhage challenge appear to be very similar across treatment arms however with slightly less variability for SB705498 3% compared to placebo.

The adjusted means for each treatment arm for each challenge from the mixed effects model for the average itch intensity (Weighted Mean Over 15 Mins) can be seen in Table 4 and the comparisons for each compared to placebo can be seen in Table 5.

**Table 4:** Adjusted Means for Average Itch Intensity.

Challenge	Treatment	N	n	Adjusted means (Std Err)	95% Confidence Interval
Cowhage	Placebo	10	9	21.62 (2.838)	(15.32, 27.93)
	SB705498 3%	10	10	20.99 (2.805)	(14.72, 27.25)
Histamine	Placebo	10	10	23.67 (3.999)	(14.86, 32.48)
	SB705498 3%	10	10	19.02 (3.999)	(10.21, 27.83)

**Table 5:** Treatment Comparison for Average Itch Intensity.

	Challenge	Comparison	Adjusted Mean Difference (std Err)	95% Confidence Interval	Probability Difference^1^		
					<0	<−5	<−10
Weighted mean over 15 mins	Cowhage	SB705498 3% - Placebo	−0.64 (1.307)	(−3.71, 2.44)	0.68	<0.01	<0.01
	Histamine	SB705498 3%- Placebo	−4.65 (2.514)	(−10.44, 1.15)	0.95	0.45	0.03

1 Posterior Probability the treatment difference is less than the stated number. This was calculated assuming non-informative priors.

The average difference between SB705498 3% and placebo for the cowhage challenge was -0.64 (95% C.I. (−3.71, 2.44)) indicating a small average reduction in average itch intensity for those on SB705498. The probability based on the observed data that the true difference is less than zero on the VAS, i.e. any beneficial effect compared with placebo, was 68% and the probability for a 5 point improvement for SB705498 3% is less than 1%.

The histamine challenge showed on average a −4.65 (95% C.I. (−10.44, 1.15)) reduction in average itch intensity (Weighted Mean Over 15 Mins) for SB705498 3% compared to placebo. For histamine the probability based on the observed data that the true difference is less than zero on the VAS, i.e. any beneficial effect compared with placebo, was 95% and the probability for a 5 point improvement for SB705498 3% is 45%.

#### Itch Intensity (Weighted Mean over Itch Period)

Itch Intensity over the itchy period, defined as the time from itch onset until time to zero itch, or the end of the 15 min period was calculated. The average difference between SB705498 3% and placebo for the cowhage challenge over the itch period was −2.08 (95% C.I. (−6.21, 2.04)) again indicating a small reduction in average itch intensity for those on SB705498 (See Table 6). The probability based on the observed data that the true difference is less than zero on the VAS, i.e. any beneficial effect compared with placebo, was 86% and the probability for a 5 point improvement for SB705498 3% was 7%. There was a small statistical difference on the histamine data as this showed a point change of −4.71 (95% C.I.−9.12, −0.31) however was not deemed clinically significant (See Table 7).

**Table 6:** Adjusted Means for Weighted Mean over Itch Period.

Challenge	Treatment	N	n	Adjusted means (Std Err)	95% Confidence Interval
Cowhage	Placebo	10	9	35.54 (1.639)	(32.05, 39.03)
	SB705498 3%	10	10	33.45 (1.546)	(30.15, 36.76)
Histamine	Placebo	10	10	31.02 (3.265)	(23.81, 38.24)
	SB705498 3%	10	10	26.31 (3.265)	(19.09, 33.52)

**Table 7:** Treatment Comparisons for Weighted Mean over Itch Period.

	Challenge	Comparison	Adjusted Mean Difference (std Err)	95% Confidence Interval	Probability Difference^1^		
					<0	<−5	<−10
Weighted mean over itch period	Cowhage	SB705498 3% - Placebo	−2.08 (1.786)	(−6.21, 2.04)	0.86	0.07	<0.01
	Histamine	SB705498 3% - Placebo	−4.71 (1.910)	(−9.12, −0.31)	0.98	0.44	0.01

1 Posterior Probability the treatment difference is less than the stated number. This was calculated assuming non-informative priors.

#### Secondary Endpoints

Table 8 shows the results of the summary statistics calculated for each secondary endpoint over the 15 minute period during which itch was recorded. Consistent results were seen across all endpoints.

**Table 8:** Summary Statistics for Secondary Endpoints.

	Challenge	Treatment	N	n^1^	Mean	Standard Deviation	Median	95% Confidence Interval
**Time to Itch onset (seconds)**	Cowhage	Placebo	10	9	136.2	78.65	169.7	75.8, 196.7
		SB705498 3%	10	10	176.1	23.44	185.0	159.3, 192.8
	Histamine	Placebo	10	10	175.5	18.78	169.0	162.1, 188.9
		SB705498 3%	10	10	180.1	25.40	172.5	162.0, 198.3
**Time to zero Itch (seconds)**	Cowhage	Placebo	10	7	518.8	138.15	483.0	391.1, 646.6
		SB705498 3%	10	8	548.0	122.18	500.4	445.9, 650.2
	Histamine	Placebo	10	8	657.7	134.71	658.0	545.0, 770.3
		SB705498 3%	10	8	644.0	165.97	620.9	505.3, 782.2
**Duration of Itch (Seconds)**	Cowhage	Placebo	10	7	343.7	147.44	307.8	207.3, 480.1
		SB705498 3%	10	8	369.0	113.60	330.9	274.0, 464.0
	Histamine	Placebo	10	8	486.7	132.72	479.4	375.7, 597.6
		SB705498 3%	10	8	461.1	166.07	440.7	322.2, 599.9
**Time to peak Itch (seconds)**	Cowhage	Placebo	10	9	277.9	103.61	216.3	198.2, 357.5
		SB705498 3%	10	10	234.2	33.81	230.2	210.0, 258.4
	Histamine	Placebo	10	10	336.0	257.05	223.5	152.1, 519.9
		SB705498 3%	10	10	277.7	109.62	218.9	199.2, 356.1
**Peak Itch Intensity**	Cowhage	Placebo	10	9	88.8	8.81	86.0	82.0, 95.6
		SB705498 3%	10	10	87.2	14.05	87.5	77.1, 97.3
	Histamine	Placebo	10	10	72.3	19.39	78.9	58.4, 86.2
		SB705498 3%	10	10	65.4	16.51	60.0	53.6, 77.2
**Duration of Peak Itch (Milliseconds)**	Cowhage	Placebo	10	9	172.9	141.32	118.5	64.3, 281.6
		SB705498 3%	10	10	204.2	175.64	164.2	78.5, 329.8
	Histamine	Placebo	10	10	154.0	211.41	44.7	2.8, 305.2
		SB705498 3%	10	10	166.8	192.71	105.2	29.0, 304.7

1 Some of these values are not 100% of the population because time to zero itch, for example, was only recorded if the subject returned to zero itch. Likewise duration of itch was only recorded if time to zero itch had occurred.

## Discussion

No clinically significant drug related AE's or any SAE's were reported from either Part A or Part B of the study.

### Part A

Capsaicin is a selective and potent exogenous agonist for the TRPV1 receptor, application of which will produce a flare on the skin. We were able to see a reduction in flare with all three doses of the TRPV1 antagonist SB705498 cream indicating TRPV1 receptor engagement was achieved.

There was no clear dose response observed for each of the three doses at either timepoint for the capsaicin challenge, however the 3% dose performed better - showing on average a 20% better response then the 5% cream at 35 mins post challenge (p-values  = 0.2927). The difference between the 3 and 5% dose was likely due to saturation with the 3% dose having the greatest amount of drug in solution.

The 3% SB705498 cream produced the largest reduction in area of flare in comparison to the other two doses at the 35 minute time point and therefore was selected to take forward to Part B. All of the doses, including the placebo had smaller areas of flare at 60 minutes. This may have been a result of the decrease in effect of the capsaicin cream at that time point, which has been observed in other studies [27].

The main objective, to show engagement of the TRPV1 receptor mechanism was achieved.

### Part B

Based on the available literature a clinically effective treatment would be expected to be associated with a 20 point change in the itch COVAS score compared with placebo.

TRPV1 has a proven role in itch and in particularly histamine induced itch. As histamine induces itch by activating the TRPV1 signalling pathway and certain pruritogens, including ATP, lipoxygenase products, acids and prostaglandins, are known to potentiate TRPV1 activity on sensory neurons. Therefore a topical TRPV 1 receptor antagonist that can block the TRPV 1 receptors located at keratinocytes and intraepidermal nerve fibres would be a good candidate drug for itch. However no differentiation was observed in the average itch intensity between the 3% cream and placebo following cowhage challenge (0.64 difference compared to placebo with 95% confidence intervals). The 3% SB705498 treatment in some subjects showed individually notable responses with respect to reduction in pruritus caused by histamine but as a cohort there was no clinically significant difference in the results compared to placebo (4.65 for histamine with 95% confidence intervals).

The time to itch onset data showed an increase in the mean time to itch onset of 39.9 seconds for the cowhage challenge and 4.6 seconds for the histamine challenge. This delay in time to itch onset is not thought to be enough to have any significant impact on the itch scratch cycle in AD.

Though the results were conclusive there were possible limitations to the study. Only male subjects were included in the study. The itch recordings on the COVAS score were subjective and subject to variation. Part B looked at the 10 best responders and hence the results should have been skewed towards those individuals most likely to show a response rather than representative of the general population. SB705498 is relatively insoluble and hence the study may have been limited by the formulation. Though a reduction in flare was observable indicating TRPV1 antagonism had taken place we may not have achieved the maximum effect on TRPV1 receptors.

The TRPV1 antagonist PAC-14028 has been shown to be effective in the attenuation of inflammation and pruritus associated with atopic dermatitis in mice (for summary see [28], [29]). PAC-14028 belongs to a novel class of non-vanilloid TRPV1 antagonists with a cinnamoyl background and hence the properties of the molecule may make it suitable for an anti-pruritic treatment.

## Conclusions

Overall the data shows that a topical formulation of 3% SB705498 cream was clinically well tolerated, with no clinically significant drug related AE's or any SAE's reported from either Part A or Part B of the study. As demonstrated by the reduction in flare following capsaicin challenge engagement of the mechanism and target specific pharmacodynamic activity in humans was seen. However engagement of the mechanism did not translate into what is believed to be a clinically significant effect on pruritus induced by either cowhage or histamine in comparison to placebo. An effect was noted with regard to the histamine challenge but this was not felt to be clinically significant. It may be possible that application of histamine by the skin prick method might have lead to recruitment of nerve endings not reached by SB705498 and hence if a formulation with a greater skin penetrance was used a clinically significant impact on the itch induced by histamine may have been seen.

The biology of itch is regulated by a number of highly complex pathways, and information was obtained during the experimental medicine study which indicated that TRPV1 receptor appears to have a minor effect on histamine-mediated pruritus. These findings indicate there are other mechanisms, yet to be elucidated, involved in the initiation and relief of histamine-independent itch induction [30].

The implications of these results for further development of SB705498 as an effective treatment for AD indicate that whilst we can see that the topical formulation of SB705498 does engage the mechanism and has shown some reduction in histaminergic itch, this reduction is not thought to be significant enough to likely elicit a beneficial treatment for dermatitis type diseases that involve significant pruritus.

## Supporting Information

Protocol S1 **VRD115246_Published Protocol Amendment 1.**
(PDF)

Checklist S1 **Consort Checklist TrpV1 Clinical Paper.**
(DOC)
