# Supplementary material for: A Randomised Trial Evaluating the Effects of the TRPV1 Antagonist SB705498 on Pruritus Induced by Histamine, and Cowhage Challenge in Healthy Volunteers
Source: PLoS One. 2014 Jul 21;9(7):e100610. doi: 10.1371/journal.pone.0100610 (PMC4105653; doi:10.1371/journal.pone.0100610)
Supplement: Protocol S1 — VRD115246_Published Protocol Amendment 1. (PDF) [file pone.0100610.s001.pdf]

Division: Worldwide Development
Retention Category: GRS019
Information Type: Protocol Amendment

**Title:**	A two part randomized, double-blind, placebo controlled study to investigate the effects of topical doses of SB705498 on capsaicin, histamine, and cowhage responses in healthy volunteers

Compound Number: SB-705498

Effective Date: 25-MAY-2012

Protocol Amendment Number: 01

Subject: Itch, healthy volunteers, SB705498, Histamine, Cowhage, Capsaicin, TRPV1, Pruritus, Clinical, Atopic Dermatitis.

Author: [redacted]

Revision Chronology:

2011N114926_00	2012-APR-11	Original
2011N114926_01	2012-MAY-25	Amendment No.: 01 Minor change to the QTc withdrawal criteria to retain the consistency with the inclusion criteria

Copyright 2012 the GlaxoSmithKline group of companies. All rights reserved. Unauthorised copying or use of this information is prohibited.

SPONSOR SIGNATORY:

[redacted]

25/MAY/2012
Date

SPONSOR/MEDICAL MONITOR INFORMATION PAGE

Medical Monitor and Sponsor Contact Information:

Role	Name	Day Time Phone Number	After-hours Phone/Cell/ Pager Number	Fax Number	GSK Address
[redacted]	[redacted]	[redacted]	[redacted]	[redacted]	[redacted]
[redacted]	[redacted]	[redacted]	[redacted]	[redacted]	
[redacted]	[redacted]	[redacted]	[redacted]	[redacted]	

Sponsor Registered Address:

[redacted]

In some countries, the clinical trial sponsor may be the local GlaxoSmithKline affiliate company (or designee). If applicable, the details of the alternative Sponsor and contact person in the territory will be provided to the relevant regulatory authority as part of the clinical trial application.

Regulatory Agency Identifying Number(s): EudraCT number 2012-000182-20

INVESTIGATOR PROTOCOL AGREEMENT PAGE

I confirm agreement to conduct the study in compliance with the protocol, as amended by this protocol amendment.

Investigator Name:		
Investigator Address:		
Investigator Phone Number:		
Investigator Signature		Date

TABLE OF CONTENTS

PAGE

ABBREVIATIONS 9

1. INTRODUCTION 11
   1.1. Background 11
   1.2. Pre-Clinical Findings 12
   1.3. Clinical Efficacy 13
   1.4. Clinical Safety 13
   1.5. Rationale 15
      1.5.1. Study Rationale 15
      1.5.2. Capsaicin 15
      1.5.3. Cowhage 15
      1.5.4. Histamine 16
   1.6. Dose Rationale 16
      1.6.1. SB705498 16
      1.6.2. Capsaicin 16
      1.6.3. Cowhage 17
      1.6.4. Histamine 17
   1.7. Summary of Risk Management 17
      1.7.1. SB705498 17
      1.7.2. Capsaicin 18
      1.7.3. Histamine 19
      1.7.4. Cowhage 19
      1.7.5. Other study procedures 20

2. OBJECTIVE(S) 20
   2.1. Part A 20
      2.1.1. Primary Objectives for Part A 20
      2.1.2. Secondary Objectives for Part A 20
   2.2. Part B 20
      2.2.1. Primary Objectives Part B 20
      2.2.2. Secondary Objectives for Part B 20

3. ENDPOINT(S) 20
   3.1. Part A 20
      3.1.1. Primary Endpoint Part A 20
      3.1.2. Secondary Endpoint Part A 20
      3.1.3. Primary Endpoints Part B 21
      3.1.4. Secondary Endpoints Part B 21

4. INVESTIGATIONAL PLAN 21
   4.1. Study Design 21
      4.1.1. Discussion of Design 21
      4.1.2. Design of Part A 21
      4.1.3. Part B 22
      4.1.4. Cowhage/ Histamine Challenge 23
   4.2. Treatment Assignment 24
   4.3. Investigational Product and Other Study Treatment Dosage/Administration 26

4.4. Dose Adjustment/Stopping Criteria 26
4.4.1. Dose Adjustment/Stopping Safety Criteria 27
4.4.1.1. Liver Chemistry Stopping Criteria 27
4.4.1.2. QTc Withdrawal Criteria 27
4.4.2. Stopping Criteria 28
4.5. Time and Events Table 29
4.5.1. Part A 29
4.5.2. Capsaicin challenge detailed schedule- Part A 30
4.5.3. Part B 31

5. STUDY POPULATION 32
5.1. Number of Subjects 32
5.2. Eligibility Criteria 32
5.2.1. Inclusion Criteria 32
5.2.2. Exclusion Criteria 33
5.2.3. Other Eligibility Criteria Considerations 34

6. DATA ANALYSIS AND STATISTICAL CONSIDERATIONS 35
6.1. Hypotheses and Treatment Comparisons 35
6.1.1. Part A 35
6.1.2. Part B 35
6.2. Sample Size Considerations 35
6.2.1. Sample Size Assumptions 35
6.2.2. Sample Size Sensitivity 36
6.2.3. Interim Analysis 36
6.2.4. Final Analyses 36
6.2.4.1. Safety Analyses 37
6.2.4.2. Pharmacokinetic Analyses 37
6.2.4.3. Pharmacokinetic/Pharmacodynamic Analyses 37

7. STUDY ASSESSMENTS AND PROCEDURES 37
7.1. Demographic/Medical History Assessments 38
7.2. Safety 38
7.3. Challenge Agent Assessments 39
7.3.1. Flare Intensity and Area (Laser Doppler Imager) 40
7.3.2. Computerised visual analogue scale (COVAS) 40
7.4. Pregnancy 41
7.4.1. Time period for collecting pregnancy information 41
7.4.2. Action to be taken if pregnancy occurs 41
7.4.3. Action to be taken if pregnancy occurs in a female partner of a male study subject 41
7.5. Pharmacokinetics 41
7.5.1. Blood Sample Collection 41
7.5.2. Sample Analysis 42

8. LIFESTYLE AND/OR DIETARY RESTRICTIONS 42
8.1. Contraception Requirements 42
8.1.1. Female Subjects 42
8.1.2. Male Subjects 43
8.2. Meals and Dietary Restrictions 43
8.3. Caffeine, Alcohol, and Tobacco 43
8.4. Activity 43

9. CONCOMITANT MEDICATIONS AND NON-DRUG THERAPIES 44
9.1. Permitted Medications 44
9.2. Prohibited Medications 44
9.3. Non-Drug Therapies 44

10. COMPLETION OR EARLY WITHDRAWAL OF SUBJECTS 44
10.1. Subject Completion 44
10.2. Subject Withdrawal Criteria 44
10.3. Subject Withdrawal Procedures 44
10.3.1. Subject Withdrawal from Study 45
10.3.2. Subject Withdrawal from Study Treatment 45
10.4. Treatment After the End of the Study 45
10.5. Screen and Baseline Failures 45

11. STUDY TREATMENT 45
11.1. Blinding 45
11.2. Packaging and Labeling 46
11.3. Preparation/Handling/Storage/Accountability 46
11.4. Assessment of Compliance 47
11.5. Treatment of Study Treatment Overdose 47

12. CHALLENGE AGENTS 47

13. ADVERSE EVENTS (AE) AND SERIOUS ADVERSE EVENTS (SAE) 48
13.1. Definition of Adverse Events 48
13.2. Definition of Serious Adverse Events 49
13.3. Method of Detecting AEs and SAEs 50
13.4. Recording of AEs and SAEs 51
13.5. Evaluating AEs and SAEs 51
13.5.1. Assessment of Intensity 51
13.5.2. Assessment of Causality 52
13.6. Follow-up of AEs and SAEs 52
13.7. Prompt Reporting of SAEs to GSK 52
13.8. Regulatory Reporting Requirements for SAEs 53

14. LIVER CHEMISTRY FOLLOW-UP PROCEDURES 53

15. STUDY CONDUCT CONSIDERATIONS 55
15.1. Posting of Information on Clinicaltrials.gov 55
15.2. Regulatory and Ethical Considerations, Including the Informed Consent Process 55
15.2.1. Urgent Safety Measures 56
15.3. Quality Control (Study Monitoring) 56
15.4. Quality Assurance 56
15.5. Study and Site Closure 57
15.6. Records Retention 57
15.7. Provision of Study Results to Investigators, Posting to the Clinical Trials Register and Publication 58
15.8. Data Management 58

16. REFERENCES 59

APPENDICES 62

Appendix 1: Liver Safety Algorithms 62
Appendix 2: Protocol Amendment Changes 63

ABBREVIATIONS

AE	Adverse Event
AD	Atopic Dermatitis
AUC	Area under concentration-time curve
BID	Twice a day
BMI	Body mass index
BP	Blood pressure
BUN	Blood urea nitrogen
CI	Confidence Interval
CL	Systemic clearance of parent drug
Cmax	Maximum observed concentration
Cmin	Minimum observed concentration
COVAS	Computerised visual analogue scale
$C\tau$	Pre-dose (trough) concentration at the end of the dosing interval
Ct	Last observed quantifiable concentration
CPSR	Clinical Pharmacology Study Report
CRF	Case Report Form
CRU	Clinical Research Unit
CPSSO	Clinical Pharmacology Science and Study Operations
CUC	Clinical Unit Cambridge
DB	Discovery Biometrics
DBP	Diastolic blood pressure
DMPK	Drug Metabolism and Pharmacokinetics
DNA	Deoxyribonucleic acid
ECG	Electrocardiogram
ENFs	Epidermal Nerve Fibers
FDA	Food and Drug Administration
FTIH	First time in humans
GCP	Good Clinical Practice
GCSP	Global Clinical Safety and Pharmacovigilence
GLP	Good Laboratory Practice
GSK	GlaxoSmithKline
GSB	Blobal Safety Board
hCG	Human chorionic gonadotropin
HIV	Human Immunodeficiency Virus
h/hr	Hour(s)
HR	Heart rate
IB	Investigator's Brochure
ICH	International Conference on Harmonization of Technical Requirements for Registration of Pharmaceuticals for Human Use
IEC	Independent Ethics Committee
IP	Investigational Product
IV	Intravenous
Kg	Kilogram
LFTs	Liver function tests

μg	Microgram
μL	Microliter
Mg	Milligrams
mL	Milliliter
nm	Nanometer
PAR	Protease-activating receptors
PD	Pharmacodynamic
PGx	Pharmacogenetics
PI	Principal Investigator
PK	Pharmacokinetic
PSRI	Periodic Safety Reports for Investigators
QC	Quality control
RAP	Reporting and analysis plan
SAE	Serious adverse event(s)
SAS	Statistical Analysis Software
SD	Standard deviation
SOP	Standard Operating Procedure
SPM	Study Procedures Manual
SUSAR	Suspected, Unexpected, Serious Adverse drug Reaction
Tmax	Time of occurrence of Cmax
ULN	Upper limit of normal
TRPV1	transient receptor potential vanilloid type 1
UK	United Kingdom
w/v	Mass/volume
w/w	Weight/Total weight

Trademark Information

Trademarks of the GlaxoSmithKline group of companies
NONE

Trademarks not owned by the GlaxoSmithKline group of companies
Chiron RIBA
Medoc
Moor
WinNonlin

1. INTRODUCTION

1.1. Background

Pruritus (itching) is the most common symptom of skin disease and can best be defined as an unpleasant cutaneous sensation that leads to a desire to scratch (Patel & Yosipovitch, 2011 and Yosipovitch, 2000). It can also be a troublesome symptom in systemic disease and psychiatric disorders. All human beings experience pruritus in the course of their lifetime.

Chronic itch, which lasts for longer than 6 weeks, has a profound impact on quality of life, including effects in sleep, attention, and sexual function. At present, there is no universally accepted therapy for itch.

Historically, the neuronal pathways for itch have been characterized mainly based on responses to histamine. Intracutaneous application of histamine produces intense itch and a large area of axon-reflexive vasodilation (“flare”) around the application site. Both phenomena are thought to be mediated through neuronal activity in itch-specific, mechanoinsensitive C-fibre afferents (CMi).

There are many direct mediators of itch and there may be redundant systems. Numerous publications have identified the transient receptor potential (TRP) channels (e.g. TRPV1, TRPV3, TRPA1, TRPM8) as having a key role in pruritus (for review see Bíró, 2007). Ultimately these channels are key in depolarizing itch sensing neurons independent of upstream (redundant) pathways. Blocking these channels has the potential to block the sensation. Published biological data indicates the Transient receptor potential vanilloid type 1 (TRPV1) receptor, as having a major role in pruritus and atopic dermatitis.

The TRPV1 receptor, can be activated by the TRPV1 agonist capsaicin or endogenous inflammatory mediators. The TRPV1 receptor is expressed in skin tissue including keratinocytes and peripheral sensory nerve fibres (C and Aδ) of itch.

One common disease with pruritus as a key symptom is atopic dermatitis (AD), which is frequently described as "the itch that rashes." Intensely itchy patches form. These patches can be widespread or limited to a few areas. Scratching often leads to redness, swelling, cracking, "weeping" of clear fluid, crusting, and scaling of the skin. TRPV1 has been shown to be upregulated in AD-skin lesions, and the activation of TRPV1 causes the release of proinflammatory and pruritic mediators (Steinhoff, 2004; Hutter, 2005; Imamachi, 2009). The activation of TRPV1 leads to the release of substance P and subsequently upregulates neurokinin-1 receptor (Velazquez, 2002; Hutter, 2005). Substance P can contribute to the development of neurogenic inflammation and pruritus, and neurokinin-1 receptor antagonists can suppress scratching behaviours (Ohmura, 2004; Yamaoka, 2007 and Kawana, 2007), Yun, 2011b) have shown that a novel TRPV1 antagonist (PAC-14028) can suppress scratching behaviours in a mouse model of AD, through the inhibition of TRPV1 activation. This suggests that the antipruritic effect of TRPV1 antagonist in AD may be explained, at least in part, by the suppression of substance P release.

SB705498 is a potent and selective antagonist at the TRPV1 receptor and has previously been explored for inflammatory pain, migraine and allergic and non-allergic rhinitis. Topical administration of SB705498 provides a means to examine the role of this mechanism on pruritus and hence it's therapeutic potential.

This proposed experimental medicine study will consist of two sections. Part A has been designed to establish whether the formulation is capable of delivering SB705498 to the proposed site of action, and to select an appropriate dose for Part B. Part B will examine the effect of SB705498 on pruritus initiated by a histaminergic and a non-histaminergic pathway, using two different challenge agents. This may provide important information about the role of TRPV1 in pruritus and the therapeutic potential of SB705498.

1.2. Pre-Clinical Findings

SB705498 is a TRPV1 antagonist that has demonstrated in vitro antagonist activity against cloned human receptors and when orally administered has shown pharmacodynamic activity in animal models of pain and nasal secretion.

SB705498 was well tolerated in oral, intranasal and dermal single and /or repeat dose studies in rats, mice, rabbits and dogs. There were no safety pharmacology findings of concern for clinical use, and the data from genotoxicity assessments suggest that SB705498 does not present a genotoxic hazard to humans. The target organ toxicity of SB705498 has been characterised in oral repeat dose studies up to 13 weeks in duration. Following oral dosing, target organs identified included adrenal (cortical microvesicular vacuolation or cortical hypertrophy), ovary (vacuolation of interstitial cells), testis (vacuolation of interstitial cells), and gall bladder. The changes noted in these organs are considered to be consistent with inhibition of steroid hormone synthesis and accumulation of precursors. Other findings noted following oral dosing include minor changes in haematological parameters with a correlated increase in extramedullary haemopoiesis and microscopic changes in the thyroid, mandibular lymph node, pituitary and kidney. Dermal administration is anticipated to minimise systemic exposure in the planned clinical trial and will not exceed the systemic exposures achieved in the completed oral and intranasal clinical studies.

Following dermal administration, in the rabbit non-adverse erythema and oedema was observed with microscopic findings (minimal to mild) of epidermal hyperplasia, dermal haemorrhage and dermal mixed inflammatory cell infiltrate. The NOAEL for topical application of SB705498 as Cream P was 5%. In the mouse local lymph node assay SB705498 was not a contact sensitizer following dermal administration at concentrations up to 5% (w/w) for 3 consecutive days.

Recent publications from one group of researchers (Bode, 2009 and Li, 2011), suggest that chronic blockade of the TRPV1 receptor may potentially promote skin tumorgeinesis. The promotion of mouse skin carcinogenesis is considered to be mediated through EGFR / Akt/mTOR signalling pathway. However, GSK has not been able to demonstrate any increase in the expression level of EGFR or Akt/mTOR signalling pathway in short term studies (in vitro and in vivo). The published data are not considered to have any safety implications for short term clinical studies.

SB705498 (drug substance alone) and SB705498 formulated in cream shows some absorbance of light in the 290 to 700 nm wavelength range (minor peaks at 311and 309 nm, respectively). In the whole body autoradiography study in rats, following oral administration of [^{14}C]SB705498, drug-related material was initially widely distributed. There was however no evidence of prolonged retention in pigmented or non-pigmented skin; drug related material was detectable in the eye uveal tract for up to 3 days after single dose, but was cleared by 35 days. No toxicity has been identified in the eye during repeat dose oral toxicity studies of up to 13 weeks in duration in the Sprague Dawley rat or beagle dog. Potential toxic effects on the eye and skin were assessed during these studies by ophthalmoscopy, and macroscopic and microscopic examination. The phototoxicity potential of SB705498 will be assessed prior to Phase III.

Overall, the nonclinical data are considered to support progression into the planned dermal clinical study.

1.3. Clinical Efficacy

There is no clinical data as yet for the topical TRPV1 antagonist SB705498. However there is evidence that oral SB705498 has the ability to have an effect on TRPV1 receptors in the skin and intranasal areas. In a human volunteer experimental medicine study, oral 400 mg SB705498 had a pronounced inhibitory effect on rhinorrhea & nasal congestion induced by unilateral intranasal capsaicin challenge. From protocol- VR1111611.In addition, in a human volunteer experimental medicine study, dosing with oral SB705498 led to a significant reduction of the flare induced by the TRPV1 agonist capsaicin which was correlated with plasma SB705498 exposure levels, supporting the conclusion that the reduction in flare was drug-induced and hence TRPV1 mediated. (Chizh, 2007).

1.4. Clinical Safety

Single doses of oral SB705498 between 2 and 1000 mg have been administered to 73 healthy normal subjects, 41 migraine patients and 70 subjects undergoing dental surgery.

A study with oral SB705498 in rectal hyperalgesia was planned, but due to recruitment problems was terminated at an early stage with only one participant randomised to placebo. SB705498 has also been administered intranasaly and these studies are detailed in Table 1.

Table 1 Overview of Intranasal Conducted Clinical Studies and Extent of Exposure

Study No.	Phase	Objectives/ Main Inclusion Criteria	No. Subjects / patients	Dose/Duration /Route
**Healthy Subject Studies Completed to Date**				
VR1111610	I	Single and repeat dose, intranasal SB705498 safety, tolerability and PK study in healthy subjects	14 single dose or placebo<br>30 repeat dose: 10 on each 6,12 mg & placebo	Single intranasal doses up to 12mg<br>Twice daily 14days repeat intranasal dose 6, 12mg or placebo
VR1111611	I	Single dose, oral SB705498 on capsaicin-evoked nasal reactivity	28	Single oral dose of 400mg
**IN Patient Studies Completed to Date**				
**VR1111925**	IIa	A randomised, double blind, placebo controlled study to assess the effect of intranasal single dose SB705498 on the response to intranasal capsaicin challenge in non-allergic rhinitis patients	41: 19 active 22 placebo	Single intranasal doses of 12 mg
**VR1114974**	IIa	A randomised, double blind placebo controlled, 2 way cross over study in adults with non-allergic rhinitis to evaluate the effect of once daily administration of intranasal SB705498 12mg for two weeks and the response to a chamber challenge of cold dry air	40	Intranasal 12mg for 2 weeks
**VR1111924**	IIa	A randomised, double-blind, placebo controlled, incomplete block, 3 way cross over study in subjects with allergic rhinitis to assess the effect of intranasal repeat doses of SB705498 when administered alone or in conjunction with intranasal fluticasone propionate on the symptoms of rhinitis in the Vienna allergen challenge chamber	70	Intranasal 12mg for 8 days

Overall, SB705498 has been well tolerated in the clinical studies completed to date, with a similar proportion of subjects reporting adverse events (AEs) following dosing with SB705498 and placebo. No clinically significant ECG abnormalities or clinically significant changes in vital signs, body temperature or laboratory safety values were found in any of the study participants.

1.5. Rationale

1.5.1. Study Rationale

The aim of this study is to answer important questions about the role of transient receptor potential vanilloid type I (TRPV1) in pruritus. The study will use a topical formulation of SB705498, a selective TRPV1 antagonist.

In order to establish whether the topical formulation enables SB705498 to reach the site of action the TRPV1 agonist, a capsaicin challenge will be performed where capsaicin is applied to the volar forearm. Capsaicin is a highly selective and potent (low nanomolar affinity) exogenous agonist for the TRPV1 receptors present on polymodal mechano- and heat sensitive C fibres and mechano heat sensitive Aδ-fibers. These nerve fibres transmit both pain and itch. Capsaicin application will produce a flare on the skin.

In Part A three different doses of SB705498 will be tested together with a placebo formulation. Evidence that the SB705498 is reaching the site of action will be ascertained by a difference in the capsaicin induced flare on the SB705498 dosed area compared to the flare produced by the capsaicin on the placebo controlled area.

The dose of SB705498 producing the largest and/or most consistent average reduction in flare will be selected for Part B of the study. If no effect is observed in Part A then Part B will not be conducted.

In Part B of the study the effects of SB705498 on two pruritogens (cowhage and histamine) which produce itch induction by two different mechanisms will be assessed.

1.5.2. Capsaicin

When the TRPV1 receptor is activated by capsaicin, it may open transiently and initiate depolarization mediated by the influx of sodium and calcium ions. In the nociceptive sensory nerves which selectively express TRPV1 (mostly C- and some Aδ-fibres), depolarization results in action potentials, which propagate into the spinal cord and brain, and may be experienced as warming, burning, stinging, or itching sensations.

1.5.3. Cowhage

The cowhage spicule delivers to the skin a cysteine protein called mucunain that has been shown to be capable of activating the protease-activating receptors PAR2 and PAR4 [Reddy, 2008b]. PAR2 receptors have been implicated in the pathophysiology of itch and of AD. Cowhage stimulates nerve fibres distinct from those activated by histamine - C polymodal nociceptors which can transmit itch as well as other mechanical and noxious stumuli. [Namer, 2008; Schmelz, 2010].

1.5.4. Histamine

Histamine has been used for many decades as the experimental pruritogen of choice in numerous studies. [Melton, 1950; Ishiuji, 2009] In chronic pruritic disease the histaminergic pathway does not appear to be the most relevant pathway. However potent histamine H1 and H2 antagonists such as Doxepin have been shown to reduce pruritus significantly in a number of pruritic diseases such as AD, lichen simplex chronicus, contact dermatitis and nummular dermatitis, so histamine does play a role [Drake, 1994, Drake, 1995].

An understanding of the impact of SB705498 on pruritus initiated by cowhage or histamine may support the exploration at later stages of pruritic indications.

1.6. Dose Rationale

1.6.1. SB705498

SB705498 will be administered topically as a cream, at strengths of 1, 3 and 5% (w/w). Typically, 0.5 g of cream will be applied on each application, meaning the doses of SB705498 received will be 5, 15 and 25 mg.

Previously, SB705498 has been administered both orally up to 1000 mg and intranasally up to 12 mg BID, single and repeat dose. Following 14-days repeated intranasal SB705498 administration at a dose of 12 mg given BID 12 h apart (total daily dose 24 mg), the arithmetic mean systemic exposure (AUC(0-24h)) was 3.91 µg.h/mL (95% CI: 1.53 - 7.16 µg.h/mL) (n=10) and the arithmetic mean Cmax was 0.245 µg/mL (95% CI: 0.168 - 0.322 µg/mL) (n=10).

As the maximum dose likely to be administered topically in this study will be 50 mg (over 2 days for Part B and over a number of limited sites on the skin), the maximum systemic exposures which could be achieved are expected to be substantially lower than those achieved previously on oral administration. As a result, no drug related adverse effects are expected.

The criteria for the choice of dose for Part B will be data driven. The data collected through the Laser Doppler Imaging will be analysed and dose selection will include the data derived from the flare determined by the LDI on both placebo and also between dose levels.

1.6.2. Capsaicin

Previous studies using 0.5ml of a 0.075% capsaicin cream (which equates to around 37.5µg), showed a reproducible response of flare to capsaicin at doses of around 30µg on the forearm when an oral dose of the TRPV1 antagonist SB705498 was administered [Chizh, 2007];and [Wang, 2010]

In a previous GSK study, a unilateral, intranasal challenge model using incremental doses (0.25 - 50 µg) of capsaicin in healthy subjects was evaluated [GlaxoSmithKline Document Number GH2007/00009/00.. Study ID VR1111611]. There was a dose-dependent increase in the weight of nasal secretions with increase in capsaicin dose. The maximum induction of nasal secretions was achieved in response to challenge with 50µg of capsaicin (Emax), whilst the dose of capsaicin that led to half maximal response (ED_{50}) was 9.43 µg (CV: 26%). We therefore believe that an appropriate dose for this study will be 0.5mL of a 0.075% capsaicin cream (37.5µg).

1.6.3. Cowhage

The number of spicules and method of application of the cowhage spicules is based on methodology used in similar studies of itch [Papoiu, 2011]. Around 30-35 cowhage spicules will be applied to a 3x3cm square area on the skin of the forearm and gently rubbed in to facilitate contact. Personal communication from Professor Gil Yosipovitch, based on his previous research, indicates that this number of spicules produce a reproducible sensation of itch.

1.6.4. Histamine

Delivery of histamine will be done according to the skin prick method using 1% histamine solution [Kofler, 2011]. This is a widely accepted method used in allergy clinics to test for histamine sensitivity; causing pruritus, skin flare and a skin wheal.

The formulation and dose of histamine is based on methodology used in similar studies of itch [Papoiu, 2011].

1.7. Summary of Risk Management

1.7.1. SB705498

SB705498 (drug substance alone) and SB705498 formulated in cream shows some absorbance of light in the 290 to 700 nm wavelength range (minor peaks at 311and 309 nm, respectively). which may indicate some potential risk associated with exposure to UV rays. There are several approved topical drugs that absorb within the visible light range. This includes topical calcipotriene/calcipotriol, used to treat psoriasis and topical retinoids approved to treat acne, psoriasis, and wrinkles. In order to minimize any potential photosensitivity risk it is advised that subjects should avoid excessive exposure to either natural or artificial sunlight (including tanning booths, sun lamps, etc.). Subjects should use sunscreen of at least SPF30 and wear protective clothing when outdoors for a week after the last dose of SB705498. Subjects should also avoid photosensitizing drugs (e.g, tetracycline, thiazides, fluoroquinolones, phenothiazines, or sulfaonamides). Subjects unwilling to adhere to this will not be included in the study according to the inclusion/exclusion criteria.

The anticipated systemic exposures associated with the proposed topical doses have been well tolerated in previous studies and are not expected to result in systemic adverse effects.

Table 2 shows the potential systemic exposure and the corresponding safety margins following dermal administration calculated assuming 10, 20, 60 and 100% bioavailability at maximum daily doses of 10, 30 and 50mg, respectively. The highest observed systemic exposure (AUC) seen following BID administration of SB705498 at 12 mg (i.e. total daily dose of 24mg for 14d) for 14 days in the intranasal clinical study has been used to calculate dermal systemic exposures, assuming that the systemic exposure (AUC) scaling is linear. Safety margins have been calculated for the estimated AUCs relative to the previously defined GSK Global Safety Board (GSB) safety limit of 26 μg.h/mL in a 24 h period, and therefore represent worst case scenario. In the clinical protocol we have calculated, as a worst case scenario, the maximum dose likely to be administered as 45 mg over 2 days (Part A), and the maximum daily dose will be 50 mg (Part B). It is anticipated that the maximum systemic exposure following dermal administration of the maximum daily dose (50 mg) will be substantially lower than those achieved in the intranasal 14 day clinical study.

Based on previous experience with topical application of drugs and the amount of body surface area to be treated, it would be expected that <10% of the drug applied would be measured in the plasma.

Table 2 Potential systemic exposures to SB705498

Cream strength (% w/w)	Max. Daily dose (mg)	% F	AUC (μg.h/mL)	fold safety cover
5	50 (2x 25mg)	100	15.8	1.52
		60	9.50	2.53
		20	1.90	12.6
		10	0.190	126
3	30 (2x15mg)	100	9.50	2.53
		60	5.70	4.21
		20	1.14	21.1
		10	0.114	211
1	10 (2x5mg)	100	3.17	7.58
		60	1.90	12.6
		20	0.38	63.2
		10	0.04	632

1.7.2. Capsaicin

Topical application of capsaicin in humans initially activates cutaneous small-diameter sensory fibres, resulting in a burning sensation, erythema, and enhanced sensitivity to noxious and innocuous stimuli. However, repeated topical applications of capsaicin lead to desensitization and degeneration of epidermal nerve fibers (ENFs), which are predominantly C-fibre nociceptors. Three weeks of frequently applied low concentration (0.075%) topical capsaicin results in 80% reduction in ENF density and moderate thinning of the subepidermal neural plexus. The effect is reversible, and regeneration of

ENFs corresponds with return of the ability to detect painful sensations [Nolano, 1999]. Following capsaicin exposure, sensations mediated by non-TRPV1-expressing cutaneous nerve endings are expected to remain unaltered. [Bley, 2010] The subjects will be exposed to low levels of capsaicin for a short period of time and therefore we do not expect to see any desensitization or degeneration of ENFs.

Any irritation caused by capsaicin can be reduced by washing the area well with cold water. If necessary, the subject will be withdrawn from the treatment but will be followed until resolution of the adverse event.

1.7.3. Histamine

Histamine is a pleiotropic endogenous biologically active amine that is widely distributed in the body and acts on multiple organ systems. Histamine is a mediator of immune and inflammatory reactions. Histamine is formed de novo during decarboxylation of histidine by histidine decarboxylase (histamine forming capacity or HFC) and is stored in the cytoplasmic granules of mast cells and basophils. Histamine synthesis, storage and release from endogenous pools are influenced by a variety of conditions including stress, circadian rhythms, drugs and allergens.

Endogenous histamine acts on a large variety of different cell types including smooth muscle, neurons, endocrine and exocrine cells, blood cells and cells of the immune system. Histamine covers its multiple biological actions via one of several receptors including H1, H2 and H3 and most recently, H4 receptors. The receptors have been detected in mammalian brain, respiratory tract, genito-urinary system and vascular system, as well as on several types of leukocytes and haematopoietic cells. The principal receptors throughout the body are the H1 and H2 receptors.

Histamine will be applied according to the skin prick method using 1% histamine solution.

Following application of histamine, if pruritus continues to be an issue an antihistamine or other appropriate treatment could be administered at the discretion of the PI. If necessary, the subject will be withdrawn from the treatment but will be followed until resolution of the adverse event.

1.7.4. Cowhage

Proteinase activated receptors 2, PAR2 (Steinhoff, 2003), is activated exogenously by the spicules of cowhage, a tropical plant which represents a particularly valuable experimental itch inducer (Papoiu, 2011). Upon skin contact, cowhage spicules release mucunain, a cysteine protease that serves as a ligand for the PAR2 receptors [Reddy, 2008a] and elicits a strong sensation of “itch without a flare” that lasts for several minutes ([LaMotte, 2009] and [Sikand, 2009]).

Following application of Cowhage if itch is still an issue the spicules will be removed from skin using adhesive tape.

If pruritus continues to be an issue after removal of the spicules, an appropriate treatment could be administered at the discretion of the PI. If necessary, the subject will be withdrawn from the treatment but will be followed until resolution of the adverse event.

1.7.5. Other study procedures

Other study procedures are routine and are not expected to represent a significant safety risk to participants.

2. OBJECTIVE(S)

2.1. Part A

2.1.1. Primary Objectives for Part A

- To assess whether any of the three doses of SB705498 are able to adequately reach the target activated by the TRPV1 agonist capsaicin

2.1.2. Secondary Objectives for Part A

- Evaluate the safety and tolerability during application of the 3 doses (1, 3 and 5%) of SB705498 compared to placebo.

2.2. Part B

2.2.1. Primary Objectives Part B

- Assess the affect of SB705498 on itch intensity induced by challenge agents (cowhage and histamine) on the 0 to 100 Computerised Visual Analogue Scale (COVAS).

2.2.2. Secondary Objectives for Part B

- Assess the duration of itch intensity after stimulation with challenge agent.

3. ENDPOINT(S)

3.1. Part A

3.1.1. Primary Endpoint Part A

- Measurement of the area of flare induced by capsaicin as assessed by Laser Doppler Imaging (LDI)

3.1.2. Secondary Endpoint Part A

- Safety and tolerability as assessed through vital sign measurements, ECG, Clinical Laboratory data and Adverse Events.
- Intensity of flare induced by capsaicin as assessed by LDI

3.1.3. Primary Endpoints Part B

- Average itch over maximum of 15 minutes post application of challenge agent (analysis of weighted means) on 0 to 100 COVAS scale.

3.1.4. Secondary Endpoints Part B

- Peak itch intensity on 0 to 100 COVAS scale
- Duration of itch from time of challenge

4. INVESTIGATIONAL PLAN

4.1. Study Design

4.1.1. Discussion of Design

This study will be conducted as a single-centre, randomised, double-blind, placebo controlled study. This study is a double blind study where the patient, investigator and site team, i.e. clinical unit staff remain blinded to the treatment. However the GSK study team not involved with study assessments will be unblinded. The GSK study team will view unblinded data from Part A to determine whether the study should progress to Part B and what the selected dose should be. The investigator and site staff will remain blinded throughout both parts of the study. An on-site unblinded pharmacist or other authorised site staff will be utilised to dispense the study medication.

A target of a maximum of 16 subjects will be recruited into Part A of the study, 10 of the best available responders will be invited to participate in Part B. If data from 10 subjects is unable to be obtained, further subjects from Part A may be asked to participate in Part B or new subjects may be enrolled to participate in Part B only, providing they pass screening and challenge agent assessments (as described in screening assessments for Part A). Male and female subjects will be eligible for screening if they meet the inclusion/exclusion criteria described in Section 5.2.

At screening, participants will be required to show a response to capsaicin and to both challenge agents - cowhage/histamine. The screening visit may be split over 2 days.

Those that respond to capsaicin and both challenge agents scoring greater than 40 on the 0-100 COVAS scale will be eligible for randomization into Part A and potentially Part B of the study.

4.1.2. Design of Part A

In Part A, up to 16 healthy Caucasian volunteers will be recruited to participate in the capsaicin challenge.

Subjects will receive all three active applications of 1%, 3% and 5% of SB705498 as well as a placebo in a randomised order (1, 2, 3 and 4) on four discrete 3x3cm square patches on the volar surface of forearms as shown in Figure 3. These four challenges with capsaicin will be split over 2 days.

Prior to each challenge, the topical administration of SB705498 or placebo will be left on the arm for 1 hour. Following this the capsaicin will be applied as described in Section 7 and assessments of skin blood flow (flare) will be performed using Laser Doppler Imaging (LDI). A baseline LDI scan will be performed prior to the application of the SB705498 cream and again after 1 hr once the excess cream has been wiped away. A third scan will then occur 5 minutes after capsaicin challenge (once excess capsaicin has been wiped away). A final scan will then take place 2 hours post application of the SB-705-498. This is shown in schematic format in Figure 2. We will be looking for a reduction in flare to be considered a positive study outcome. Previous research has shown oral doses of SB705498 produced a reduction in the area of capsaicin-evoked flare by ~ 35% compared with placebo, (Chizh, 2007, **132**: 132-141)

Figure 1 Schematic of Part A- the capsaicin challenge

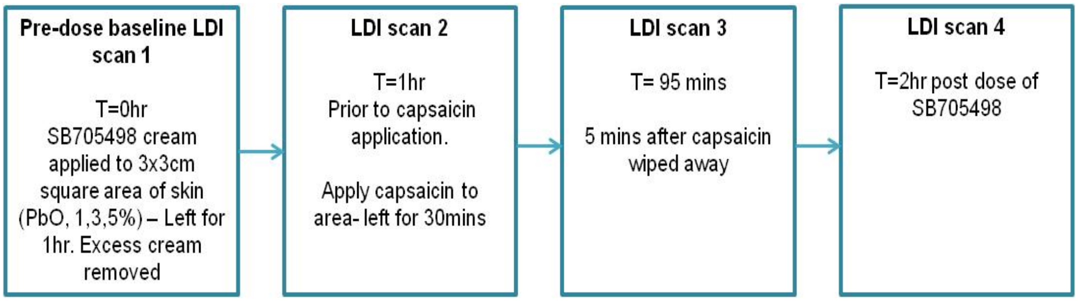

The decision to progress to Part B of the study will be made by the members of the study team after reviewing the unblinded data from Part A. A reduction in area of flare for subjects whilst receiving SB705498 compared with placebo will have to be observed on at least one of the dose strengths studied. The dose level deemed to have the largest and most consistent effect will be chosen as the dose strength studied in Part B. Details of the timepoints are listed in the Time and Events table Section 4.5.

An interim analysis may take place if the rate of recruitment is significantly slower than expected. If this is the case the GSK study team will make a decision to asses the unblinded data. If sufficient evidence is deemed to have been obtained to determine an effect of reducing the induced area of flare of the capsaicin, and the optimal dose to progress into Part B of the study is found, Part A of the study may be stopped early to start Part B. Similarly, if no effect is observed then the study may be stopped for futility.

4.1.3. Part B

Of the sixteen subjects randomised to participate in Part A, the ten subjects who show the most optimal treatment response during the capsaicin challenge compared to placebo will be asked to participate in Part B. If we are unable to obtain data from ten of the Part A subjects, we may ask the next best responders from Part A to go on to Part B or recruit new subjects to enter the study and participate in Part B of the study, providing they pass screening and challenge agent assessments.

Each volunteer will be randomised to receive the placebo and the selected topical application strength of SB705498 from Part A of the study for both the cowhage and histamine challenge. Each subject will participate in both a cowhage challenge and a histamine challenge with both treatment arms in a crossover fashion an example of a possible randomisation schedule is shown in Figure 2. Each subject will receive two doses of SB705498 over 2 days.

The cowhage challenge will be performed on a separate day to the histamine challenge.

Figure 2 Schematic of Part B – Cowhage/histamine challenge

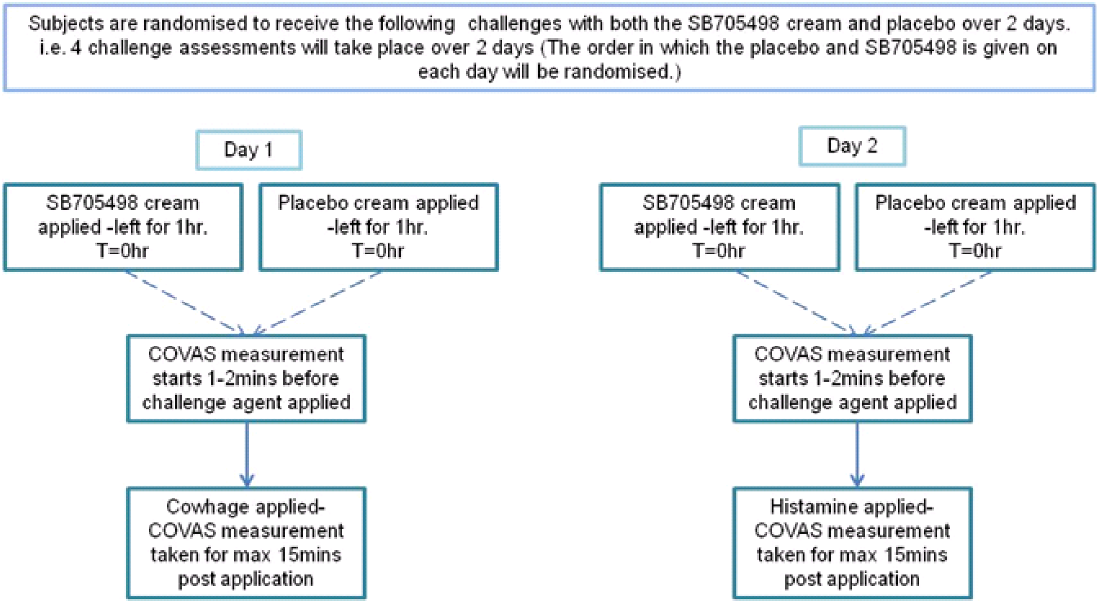

4.1.4. Cowhage/ Histamine Challenge

Subjects will be randomised to receive either the SB705498 cream or placebo treatment first. This will be applied to the volar suface of the forearm followed 1 hour later by the cowhage/ histamine stimulant. Subsequently once all itch sensations have subsided the other treatment (placebo or SB705498) will be applied to the corresponding area on the other forearm and the same stimulant (cowhage/ histamine) will be applied as before.

This whole procedure will be repeated for the remaining challenge agent, cowhage/histamine on the following day allowing time for previous itch sensations to completely subside. For each challenge agent itch intensity will be rated using the COVAS scale as previously described commencing 1-2 mins before the challenge agent is applied.

4.2. Treatment Assignment

Subjects will be assigned to a treatment sequence in accordance with the randomisation schedule generated by Clinical Statistics, prior to the start of the study, using validated internal software.

Part A

Subjects in Part A of the study will follow a Williams Design of a generalised latin square and will be randomised to one of the following sequences: 1 2 4 3, 2 3 1 4, 3 4 2 1 or 4 1 3 2 in a 1:1:1:1 ratio where one of the numbers will be placebo and the other 3 will represent each of the three active doses of SB705498.

The first number in the sequence will be applied to the first application site as shown in Figure 3. Each subsequent number will be applied to the next application site.

Part B.

Subjects in Part B of the study will be randomised to one of the following sequences: E F E F, F E F E, E F F E or F E E F in a 1:1:1:1 ratio where one of these will be placebo and the other will be SB705498. The first letter in the sequence will be applied to the first application site as shown in Figure 3. Each subsequent letter will be applied to the next application site. This ensures that the dose of SB705498 will be compared to placebo on the same area of the opposite forearm for both the histamine and cowhage test.

Figure 3 Example Schematic of application Sites

Part A

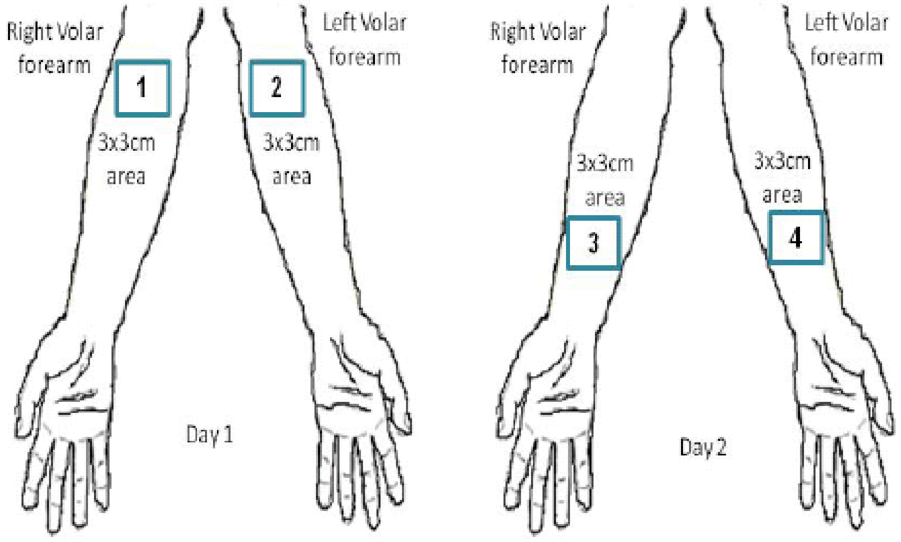

Part B

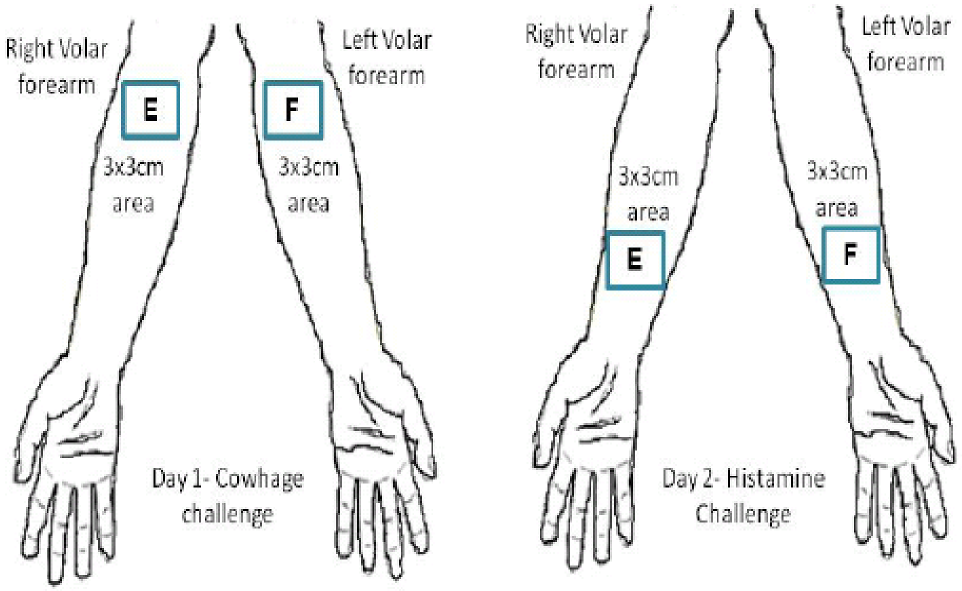

*Note: The days on which the Cowhage and Histamine challenges will take place are interchangeable.

4.3. Investigational Product and Other Study Treatment Dosage/Administration

	Study Treatment		Challenge Agent		
**Product name:**	**SB705498**	**Placebo**	**Cowhage**	**Capsaicin**	**Histamine**
**Dosage form:**	Cream	Cream	Spicules	Cream	Solution
**Unit dose strength(s)/Dosage level(s):**	1%, 3% and 5%	Placebo	30-35 spicules	0.075%	1% (10mg/mL)
**Route/ Administration/ Duration:**	Topical/cream/ 1 hour	Topical/cream/ 1 hour	Pierce Superficial layer of skin/ spicules/ 15 mins	Topical/Cream/ 30mins	Pierce Superficial layer of skin/Solution /N/A
**Dosing instructions:**	Apply cream to designated area as directed	Apply cream to designated area as directed	Gently rubbed onto designated area ofskin as directed in Section 7.3	Apply cream to designated area as directed in Section 7.3.	1% solution of histamine will be applied using the skin prick method as described in Section 7.3.
**Physical description:**	White to slightly coloured, opaque cream.	White to slightly coloured, opaque cream.	2mm brown fibres	White to slightly coloured, opaque cream.	Clear liquid
**Manufacturer/ source of procurement:**	GSK	GSK	External academic lab	Cephalon	ALK Abello
**Method for individualizing dosage:**	Use of pipette from single use container	Pipette from single use container	30-35 spicules counted out into individual single use container	Single use syringe from single use container	Individual drops with single use lancet for piercing

4.4. Dose Adjustment/Stopping Criteria

The systemic exposure associated with the proposed topical dose of SB705498 is not known prior to study start but is expected to be low. The proposed topical dose for this study is a maximum of 95 mg across both parts. Given that SB705498 has been administered as single oral doses up to 1000mg and intranasally up to 12mg twice daily for 14 days, the dose for this study has substantial safety margins and is therefore not expected to require any dose adjustment.

4.4.1. Dose Adjustment/Stopping Safety Criteria

4.4.1.1. Liver Chemistry Stopping Criteria

Liver chemistry threshold stopping criteria have been designed to assure subject safety and to evaluate liver event etiology (in alignment with the FDA premarketing clinical liver safety guidance). Study treatment will be stopped and the subject withdrawn from the study if any of the following liver chemistry stopping criteria is met in either session:

1. ALT ≥ 3xULN and bilirubin ≥ 2xULN (or ALT ≥ 3xULN and INR[1] > 1.5) NOTE: serum bilirubin fractionation should be performed if testing is available. If fractionation is unavailable, urinary bilirubin is to be measured via dipstick (a measurement of direct bilirubin, which would suggest liver injury).

1.INR testing not required per protocol and the threshold value does not apply to subjects receiving anticoagulants.

2. ALT ≥ 5xULN.
3. ALT ≥ 3xULN if associated with the appearance or worsening of rash or hepatitis symptoms (fatigue, nausea, vomiting, right upper quadrant pain or tenderness, fever, rash or eosinophilia).
4. ALT ≥ 3xULN persists for ≥ 4 weeks.
5. ALT ≥ 3xULN and cannot be monitored weekly for 4 weeks. Subjects with ALT ≥ 3xULN **and** < 5xULN **and** bilirubin < 2xULN, who do not exhibit hepatitis symptoms or rash, can continue study treatment as long as they can be monitored weekly for 4 weeks.

Refer to Section 14, Liver Chemistry Follow-up Procedures, for details of the required assessments if a subject meets any of the above criteria.

4.4.1.2. QTc Withdrawal Criteria

- QTc, QTcB, QTcF > 500 msec or uncorrected QT>600msec
- Prolongation of QTcB or QTcF by > 60 msec as compared to baseline
- If subject has underlying bundle branch block then the QTc withdrawal criteria depends on the baseline value:

**Baseline QTc value (with underlying bundle branch block)**	**QTc withdrawal criteria**
<450 ms	>500 ms

Withdrawal decisions are to be based on an average QTc value of triplicate ECGs. If an ECG demonstrates a prolonged QT interval, obtain 2 more ECGs over a brief period, and then use the averaged QTc values of the 3 ECGs to determine whether the subject should be discontinued from the study. Subjects who are withdrawn, will not return for a second session.

4.4.2. Stopping Criteria

The results from Part A will be reviewed to determine if Part B of the study will be conducted. If no effect is observed in Part A, then the study may be stopped for futility.

If more than 2 subjects are withdrawn due to severe skin reactions as result of study procedures a safety review will be triggered and no further dosing will take place pending this review. If an SAE is reported as possibly related to the study drug, the study will be put on hold pending a safety review.

4.5. Time and Events Table

4.5.1. Part A

Procedure	Screening (up to 30 days prior to Day 1)- May be split over 2 days	Study Day (per treatment arm)					Follow-up (10-14 days post-last dose)
		-30 min	Day 1 and 2				
			0	60 min	95 min	120 min	
Admission to Unit	X	X					
Informed Consent	X						
Full Physical Exam	X						
Brief Physical Exam							X
Medical/medication/ drug/alcohol history	X						
Vital signs	X	X					X
ECG	X	X					X
Urine Drug/Alcohol	X	X					
Serum β-hCG (women)- Day 1 only	X	X					X
HIV, Hep B and Hep C screen-	X						
Haem/Chem/Urinalysis tests	X						X
Administration of SB705498 cream/Placebo[4]			X				
Assess area of flare using LDI	X		X[1]	X[2]	X[3]	X	
Capsaicin challenge	X			X			
Histamine/Cowhage Challenge	X						
Assess ITCH intensity (COVAS)	X						
Adverse Event Review	X	←===→					X
Concomitant Medication Review	X	←===→					X
Pharmacokinetic sample			X	X	X	X	
Discharge							X

1. Baseline scan will be performed before application of the SB705498 cream or Placebo
2. LDI scan will be performed after application of SB705498 cream or placebo. Cream is applied, left for 1 hr and excess is then removed to allow scanning.
3. LDI scan will be performed 5 mins after the Capsaicin cream is wiped off the arm.
4. Application of the cream/placebo followed by capsaicin challenge and LDI scanning will be performed twice on each day. i.e. 4 applications over the 2 days with the 3 different doses of SB705498 and placebo.

4.5.2. Capsaicin challenge detailed schedule- Part A

The table below is based on an example randomization schedule for reference only. Each subject will be assigned an individual randomization schedule detailing which dose will be given in which order

Procedure	Study Day (per treatment arm)															
	Day 1 AM				Day 1 PM				Day 2 AM				Day 2 PM			
	0	60 min	95 min	120 min	0	60 min	95 min	120 min	0	60 min	95 min	120 min	0	60 min	95 min	120 min
Administration of SB705498 cream 1%	X															
Administration of SB705498 cream 3%					X											
Administration of SB705498 cream 5%									X							
Administration of Placebo cream													X			
SB705498/Placebo wiped away		X				X				X				X		
Assess area of flare using LDI	X[1]	X[2]	X[3]	X	X[1]	X[2]	X[3]	X	X[1]	X[2]	X[3]	X	X[1]	X[2]	X[3]	X
Capsaicin cream applied		X				X				X				X		
Capsaicin wiped away			X				X				X				X	
Pharmacokinetic sample	X	X	X	X	X	X	X	X	X	X	X	X	X	X	X	X

1. Baseline scan will be performed before application of the SB705498 cream or Placebo
2. LDI scan will be performed after application of SB705498 cream or placebo. Cream is applied, left for 1 hr and excess is then removed to allow scanning.
3. LDI scan will be performed 5 mins after the Capsaicin cream is wiped off the arm.

4.5.3. Part B

Procedure	Screening (up to 30 days prior to Day 1)[2]	Study Day (per treatment arm)								Follow-up (10-14 days post-last dose)
		Day 1				Day 2				
		-30 mins	0h	1h	1.25h	-30 mins	0h	1h	1.25h	
Admission to Unit	X	X				X				
Consent review	X									
Full Physical Exam	X									
Brief Physical Exam										X
Medical/medication/ drug/alcohol history	X									
Vital signs	X	X				X				X
ECG	X	X				X				X
Urine Drug/Alcohol	X	X				X				
Serum β-hCG (women)- Day 1 only	X	X				X				X
HIV, Hep B and Hep C screen	X									
Haem/Chem/Urinalysis tests	X									X
Capsaicin challenge	X[3]									
Assess area of flare using LDI	X[3]									
Assess ITCH intensity (COVAS)	X[3]									
Administration of SB705498 cream/Placebo[4]			X				X			
Histamine challenge[5]	X[3]							X		
Cowhage challenge[5]	X[3]			X						
COVAS scale[1]				X	X			X	X	
Adverse Event Review	X	←==→								X
Concomitant Medication Review		←==→								X

1. COVAS will be measured continuously from 1-2 mins before application of challenge agent and for a maximum time of 15 mins post application.
2. Screening assessments for Part B will only occur if the period of time between Part A screening and Part B study visit 1 exceeds 60 days.
3. To be performed for new subjects that have not taken Part in Part A.
4. Application of the cream/placebo followed by challenge agent assessment will be performed twice on each day. i.e. 4 applications over the 2 days with both SB705498 and placebo and each challenge agent.
5. The days on which the cowhage and histamine challenges are performed may be swapped according to site logistics and at the investigators discretion.

5. STUDY POPULATION

5.1. Number of Subjects

Sixteen subjects will be randomised to take part in Part A of the study. From these sixteen subjects, ten subjects who show the most optimal treatment effect compared to placebo on the selected dose will be asked to participate in Part B. If for any reason these subjects are unable to participate in Part B, other subjects from Part A with sufficient treatment effect will be asked to participate to ensure 10 subjects in Part B. Additional subjects may be enrolled into Part B to ensure 10 completers in Part B. If subjects prematurely discontinue the study, additional subjects may be enrolled as replacement subjects and assigned to the same treatment sequence at the discretion of the sponsor in consultation with the investigator.

5.2. Eligibility Criteria

5.2.1. Inclusion Criteria

A subject will be eligible for inclusion in this study only if all of the following criteria apply:

1. Healthy Caucasian volunteers aged between 18 and 65 years of age inclusive, at time of signing the informed consent. Healthy subjects are defined as individuals who are not taking any regular medication and are free from clinically significant illness or disease as determined by their medical history (including family history), physical examination, 12-lead ECG, laboratory studies, and other tests specified in this protocol.
2. Subject has the ability to read, comprehend, and record information required by protocol, and is willing and able to provide signed and dated written informed consent prior to study participation, including compliance with the requirements and restrictions listed in the consent form.
3. Non smoker who has not smoked or used nicotine containing products for a minimum period of 6 months prior to the screening visit.
4. Subject must score greater than 40 on a COVAS scale of 0-100 for itch induced by both cowhage and histamine at screening and show a flare response with capsaicin measured by LDI scan.
5. Is willing to avoid UV exposure. i.e. use of Sun beds and sunbathing for 7 days prior to screening, throughout the study and and until a week after the last dose. Subjects who require treatment with photosensitizing drugs (e.g, tetracycline, thiazides, fluoroquinolones, phenothiazines, or sulfaonamides) will be withdrawn. Subjects must also be willing to use sunscreen of SPF30 or above on the volar surface of the forearms for a week after the last dose.

6. A female subject is eligible to participate if she is of:
   - Non-childbearing potential defined as pre-menopausal females with a documented tubal ligation or hysterectomy; or postmenopausal defined as 12 months of spontaneous amenorrhea [in questionable cases a blood sample with simultaneous follicle stimulating hormone (FSH) > 40 MlU/ml and estradiol < 40 pg/ml (<147 pmol/L) is confirmatory]. Females on hormone replacement therapy (HRT) and whose menopausal status is in doubt will be required to use one of the contraception methods in Section 8.1 if they wish to continue their HRT during the study. Otherwise, they must discontinue HRT to allow confirmation of post-menopausal status prior to study enrollment. For most forms of HRT, at least 2-4 weeks will elapse between the cessation of therapy and the blood draw; this interval depends on the type and dosage of HRT. Following confirmation of their post-menopausal status, they can resume use of HRT during the study without use of a contraceptive method.]
   - Child-bearing potential and agrees to use one of the contraception methods listed in Section.8.1 Female subjects willing to participate in the study must agree to use contraception from the screening visit until 15 days post-last treatment administration.
7. Male subjects must agree to use one of the contraception methods listed in Section .8.1. For male subjects willing to participate in the study this criterion must be followed from the time of the screening visit until 15 days post-last treatment
8. BMI within the range 19.0 – 32.0 kg/m^2 (inclusive).
9. AST and ALT < 2xULN; alkaline phosphatase and bilirubin ≤ 1.5xULN (isolated bilirubin >1.5xULN is acceptable if bilirubin is fractionated and direct bilirubin.
10. QTcB or QTcF< 450 msec. Note that if the initial QTc value is prolonged, the ECG should be repeated two more times (with 5 minutes between ECG readings) and the average of the three QTc values used to determine eligibility.

5.2.2. Exclusion Criteria

Deviations from exclusion criteria are not allowed because they can potentially jeopardise the scientific integrity of the study, regulatory acceptability or subject safety. Therefore, adherence to the criteria as specified in the protocol is essential.

A subject will not be eligible for inclusion in this study if any of the following criteria apply:

1. Suffers from skin infection or inflammation of the forearm, or has other arm skin irregularities that may in the opinion of the investigator interfere with study assessments (e.g. nevi, tattoos).
2. The subject suffers from eczema, psoriasis or any other acute or chronic dermatological problem if, in the opinion of the investigator this is likely to interfere with study assessments

3. Any subject with localised sunburn in the area to be treated as part of the study.
4. History of regular alcohol/drug consumption within 6 months of the study. Regular alcohol consumption defined as: an average weekly intake of >21 units for males and >14 units for females. One unit is equivalent to 8 g of alcohol: a half-pint (~240 ml) of beer, one glass (125 ml) of wine or one (25 ml) measure of spirits.
5. A positive pre-study drug/alcohol screen at screening visit. A minimum list of drugs that will be screened for include amphetamines, barbiturates, cocaine, opiates, cannabinoids and benzodiazepines.
6. The subject has participated in a clinical trial resulting in exposure to more than four new chemical entities within 12 months prior to the first dosing day or has received an investigational product within the following time period prior to the first dosing day in the current study: 90 days, 5 half-lives or twice the duration of the biological effect of the investigational product (whichever is longer).
7. Unable to refrain from use of prescription or non-prescription drugs. Refer to Section 9 for details.
8. History of sensitivity to any of the study medications SB705498, challenge agents or components thereof or a history of drug or other allergy that, in the opinion of the investigator or GSK Medical Monitor, contraindicates their participation.
9. Where participation in the study would result in donation of blood or blood products in excess of 500 mL within a 56 day period following completion of the study.
10. Lactating females.
11. A positive pre-study Hepatitis B surface antigen or positive Hepatitis C antibody result within 3 months of screening
12. A positive test for HIV antibody.
13. Excessive caffeine drinkers (~5 or more cups a day) or the subject is unable to commit to refraining from caffeine-containing products for 24hrs prior to each assessment visit and whilst present in the unit.

5.2.3. Other Eligibility Criteria Considerations

To assess any potential impact on subject eligibility with regard to safety, the investigator must refer to the following document(s) for detailed information regarding warnings, precautions, contraindications, adverse events, and other significant data pertaining to the GSK investigational product(s) or other study treatment being used in this study:SB705498 IB [GlaxoSmithKline Document Number HM2004/00078/07]

6. DATA ANALYSIS AND STATISTICAL CONSIDERATIONS

6.1. Hypotheses and Treatment Comparisons

6.1.1. Part A

Precision Estimation

Part A of the study is designed to estimate the dose response of SB705498 on the area of induced flare caused by capsaicin as measured by a laser Doppler imaging machine. No formal hypothesis will be tested.

6.1.2. Part B

Part B of the study is designed to test for superiority. The null hypothesis for the treatment comparison will be that there is no difference between the chosen dose strength of SB705498 and placebo on the average itch intensity post application of cowhage and histamine stimulant. The alternative hypothesis will be that there is a difference. Symbolically, this is expressed as follows:

H(0): μ(test) = μ(reference)

H(1) : μ(test) $\neq$ μ(reference)

A two-sided t-test with α=0.05 will be used to test this hypothesis

6.2. Sample Size Considerations

6.2.1. Sample Size Assumptions

Part A

Based on a within subject coefficient of variation of 0.45 which was observed as the variability estimate for SB705498/001 oral dosing, [GlaxoSmithKline Document Number HM2005/00272/00], for the reduction in area of flare as caused by capsaicin challenge and assessed by LDI machine, and a sample size of 16 subjects in a crossover design provides a precision estimate of 37.7%.

The precision estimates the half width of the 95% confidence interval around the difference between treatments. It is estimated that the lower and upper bounds of the 95% confidence intervals for the ratio of SB705498: placebo will be within point estimate ± precision estimate. Hence, for the area of flare endpoint it is estimated that the 95% confidence interval will be within ± 37.7% if the within subject standard deviation is similar to that observed in SB705498/001 [GlaxoSmithKline Document Number HM2005/00272/00].

Part B

Part B of the study will be powered to detect a 20 point difference between the chosen dose of SB705498 and placebo on the 0-100 computerised VAS scale. An estimate of the variability of the computerised VAS scoring of itch after application of cowhage and histamine was obtained from an academic experiment performed by Yosipovitch et al. (Personal communication). The variability of itch on the 11 point (0-10) VAS scale for cowhage and histamine was 0.97 and 1.15 respectively. Using the higher value as our estimate of variability and with a clinically relevant difference of 2 points on the VAS scale then to have at least 90% power and with a two-sided type 1 error rate of 5%, 10 subjects are required for the crossover design.

6.2.2. Sample Size Sensitivity

Part A

The upper limit of the 95% confidence interval for the estimate of the coefficient of variation from the oral dosing study [GlaxoSmithKline Document Number HM2005/00272/00] is 0.77. Using this as the expected measure of variability then a sample size of 16 subjects in a crossover design provides a precision estimate of 66.2%.

Part B

Assuming the standard deviation is 20% higher than was previously seen in the data provided by Yosipovitch [Yosipovitch, 2000], then under the same criteria as previously specified; clinically relevant difference of 2, 90% power and a 5% two-sided type 1 error rate, then 14 subjects would be required using this crossover design.

6.2.3. Interim Analysis

Data from Part A of the study may be analysed instream if the recruitment of subjects is slower than anticipated.The GSK study team will decide if this is necessary and when it should take place. Only the primary endpoint of Part A will be analysed at the interim analysis and will be analysed as per the final analysis for Part A as discussed in Section 6.2.4.

6.2.4. Final Analyses

Part A

An appropriate dose response model will be fit to the post dose time points. We are currently unsure as to what the underlying distribution of the dose response curve is so the data will be plotted to get a visual representation of the data. After this, various models will be applied and the best fitting one will be selected.

If the data does not allow for a dose response model to be fit then area of flare will be analysed using a mixed effects repeated measures model, fitting terms for treatment, period, time, and treatment time as fixed effects and subject as a random effect. No baseline will fit in the model since no area of flare is present until the stimulant is added. Point estimates and 95% percent confidence intervals for the difference between and placebo will be constructed using an appropriate variance structure.

Model and distributional assumptions underlying all analyses will be assessed by visual inspection of residual plots. Homogeneity of variance will be assessed by plotting the raw residuals against the predicted values from the model, whilst normality will be assessed using normal probability plots. Normality of the subject effect estimates from the random term will be assessed using a normal probability plot. Alternative statistical procedures will be considered should the model assumptions be violated.

Part B

Average itch will be analysed using a mixed effects model, fitting terms for treatment, period and stimulant, as fixed effects and subject as a random effect. No baseline will fit in the model since no itch is present until the stimulant is added. Point estimates and 95% percent confidence intervals for the difference between and placebo will be constructed using the appropriate variance structure.

Model and distributional assumptions underlying all analyses will be assessed by visual inspection of residual plots. Homogeneity of variance will be assessed by plotting the raw residuals against the predicted values from the model, whilst normality will be assessed using normal probability plots. Normality of the subject effect estimates from the random term will be assessed using a normal probability plot. Alternative statistical procedures will be considered should the model assumptions be violated.

6.2.4.1. Safety Analyses

Safety data will be presented in tabular and/or graphical format and summarized descriptively according to GSK's Integrated Data Standards Library (IDSL) standards.

6.2.4.2. Pharmacokinetic Analyses

If sufficient PK data is quantifiable then appropriate analyses of the pharmacokinetic parameters, will be performed and will be documented in the RAP.

6.2.4.3. Pharmacokinetic/Pharmacodynamic Analyses

The relationship between time, concentration and the pharmacodynamic responses (as outlined in primary and secondary objectives) following topical administration of SB705498/ placebo and will be explored by graphical means where appropriate.

Further analysis using non-linear mixed effect models may be performed by CPMS if deemed appropriate

7. STUDY ASSESSMENTS AND PROCEDURES

This section lists the parameters of each planned study assessment. The exact timing of each assessment is listed in the Time and Events Table (Section 4.4). Detailed procedures for obtaining each assessment are provided in the Study Procedures Manual (SPM). Whenever vital signs, 12-lead ECGs and blood draws are scheduled for the same nominal time, the assessments should occur in the following order: 12-lead ECG, vital signs, blood draws. The timing of the assessments should allow the blood draw to occur at the exact nominal time.

The timing and number of planned study assessments, including safety, pharmacokinetic and pharmacodynamic assessments may be altered during the course of the study based on newly available data (e.g. to obtain data closer to the time of peak plasma concentrations) to ensure appropriate monitoring. The change in timing or addition of time points for any planned study assessments must be approved and documented by GSK, but this will not constitute a protocol amendment. The IRB/IEC will be informed of any safety issues that require alteration of the safety monitoring scheme. No more than 500 mL of blood will be collected over the duration of the study, including any extra assessments that may be required.

7.1. Demographic/Medical History Assessments

The following demographic parameters will be captured: date of birth, gender, race and ethnicity.

Medical/medication/alcohol history will be assessed as related to the eligibility criteria listed in Section 5.2.

7.2. Safety

Planned time points for all safety assessments are listed in the Time and Events Table (Section 4.4). Additional time points for safety tests (such as vital signs, physical exams and laboratory safety tests) may be added during the course of the study based on newly available data to ensure appropriate safety monitoring.

Physical Exams

- A complete physical examination will include assessments of the head, eyes, ears, nose, throat, skin, thyroid, neurological, lungs, cardiovascular, abdomen (liver and spleen), lymph nodes and extremities. Height and weight will also be measured and recorded.
- A brief physical examination will include assessments of the skin, lungs, cardiovascular system, and abdomen (liver and spleen).

Vital Signs

- Vital sign measurements will include systolic and diastolic blood pressure (semi-supine) and pulse rate.
- Body temperature will not be routinely monitored during the study however if any subject reports symptoms of high temperature or fever, temperature will be recorded and treated if appropriate at the investigators discretion.

Electrocardiogram (ECG)

- 12-lead ECGs (semi-supine) will be obtained at each time point during the study using an ECG machine that automatically calculates the heart rate and measures PR, QRS, QT, and QTc intervals. Refer to Section 5.2 for QTc eligibility criteria and additional QTc readings that may be necessary.

Clinical Laboratory Assessments

Haematology, clinical chemistry, urinalysis and additional parameters to be tested are listed below:

Haematology

Platelet Count	RBC Indices:	Automated WBC Differential:
RBC Count	MCV	Neutrophils
WBC Count (absolute)	MCH	Lymphocytes
Haemoglobin	MCHC	Monocytes
Hematocrit		Eosinophils
		Basophils

Clinical Chemistry

BUN	Potassium	AST (SGOT)	Total and direct bilirubin
Creatinine	Chloride	ALT (SGPT)	Uric Acid
Glucose	Calcium	GGT	Albumin
Sodium		Alkaline phosphatase	Total Protein

Routine Urinalysis

Specific gravity
pH, glucose, protein, blood and ketones by dipstick
Microscopic examination (if blood or protein is abnormal)

Other screening tests

HIV
Hepatitis B (HBsAg)
Hepatitis C (Hep C antibody -- if second generation Hepatitis C antibody positive, a hepatitis C antibody Chiron RIBA immunoblot assay should be reflexively performed **on the same sample** to confirm the result)
FSH and estradiol (as needed in women of non-child bearing potential only)
Alcohol and drug screen (to include at minimum: amphetamines, barbiturates, cocaine, opiates, cannabinoids and benzodiazepines).

7.3. Challenge Agent Assessments

Capsaicin- Approximately 0.5 mL of capsaicin cream (Axsain, 0.075% capsaicin w/w) will be applied to a 3 x 3cm square area on the volar aspect of one arm. The cream will be left on the skin for 30 min and then gently wiped off. Assessments of skin blood flow will be performed before and after capsaicin application by monitoring cutaneous blood flow using Laser Doppler imaging (LDI-2, Moor Instruments Ltd., Devon, UK). A suitable area of approximately 16 × 8 cm around the stimulation site will be scanned. The flare area (in cm^2) will be calculated from all pixels around the stimulation site in which flux values exceeded the 95% percentile (mean +2 SD) of the baseline distribution. The mean blood flow in the area of flare will also be calculated using relative flux (arbitrary units).

Only volunteers that are sensitive to capsaicin i.e. develop flare on application of 0.5 ml of Axsain (0.075% capsaicin containing cream), will be enrolled. Previous evidence from the FTIH study of SB705498 has indicated a 2.5 %- 5% failure rate according to this criterion.

Cowhage - Approximately 30-35 cowhage spicules will be counted under the microscope and applied onto a 3x3cm square area on the skin using a microtweezer. The spicules will be gently rubbed with a gloved finger for 45 seconds onto the subject's skin with a circular motion to facilitate contact. Approximately 1-2 minutes before contact with the cowhage spicules subjects will be asked to start rating their itch intensity using the computerized visual analogue scale (COVAS). Itch intensity will be rated using the 0 to 100 COVAS scale, 0 being no itch at all and 100 being the most itch imaginable. The itch intensity will continue to be recorded for 15 minutes or until an itch score of 0 (baseline) is recorded for 60 continuous seconds. Recording of pruritus will not exceed 15 minutes.

Histamine - A 1% solution of histamine will be applied using the skin prick method. [Kofler, 2011]. This is a widely accepted method used in allergy clinics to test for histamine sensitivity; causing pruritus, skin flare and a skin wheal. The formulation and dose of histamine is based on methodology used in similar studies of itch [Papoiu, 2011]. The skin prick method involves placing a drop of 1% histamine solution onto the skin and using a lancet to gently pierce the superficial layer of the skin. The excess histamine solution is then wiped away.

Itch intensity will be rated using the COVAS scale as previously described.

These challenges will take place randomly and sequentially at screening. Between itch inductions a break will be taken to allow previous itch sensations to completely subside.

7.3.1. Flare Intensity and Area (Laser Doppler Imager)

Superficial blood flow of the stimulated arm is measured repetitively by Laser Doppler Imager (LDI, Moor Instruments Ltd., Devon, United Kingdom). For this purpose, an area around the stimulation site is scanned with a typical resolution of 22,400 pixels, with each pixel representing a separate Doppler flux measurement. They are stored on hard disk and processed off-line with dedicated software (MoorLDI, Moor Instruments Ltd.). The flare area is calculated from all pixels around the stimulation site in which flux values exceeds the 95% percentile (mean + 2x S.D.) of the baseline distribution. Laser Doppler images are recorded according to Section 4.5.

7.3.2. Computerised visual analogue scale (COVAS)

The Computerised visual analogue scale (COVAS) is a psychometric continuous scale, which will be used to measure the degree of itch. COVAS will be assessed using a COVAS response box attached to a Pathway system (Medoc Ltd Israel). The scale captures continuous ratings on a 0-100 scale, where pushing the slide on the response box to the extreme left or right refers to zero (no Itch), and 100 (extreme itch) respectively.

Data is captured continually within the Pathway software (Medoc ltd) for the course of the Itch assessment time point. Data is exported within an excel spreadsheet for each individual time point for each volunteer (please refer to SPM for more detail).

7.4. Pregnancy

7.4.1. Time period for collecting pregnancy information

All pregnancies in female subjects and/or female partners of male subjects will be collected after the start of dosing and until the end of the follow up visit.

7.4.2. Action to be taken if pregnancy occurs

The investigator will collect pregnancy information on any female subject, who becomes pregnant while participating in this study. The investigator will record pregnancy information on the appropriate form and submit it to GSK within 2 weeks of learning of a subject's pregnancy. The subject will also be followed to determine the outcome of the pregnancy. Information on the status of the mother and child will be forwarded to GSK. Generally, follow-up will be no longer than 6 to 8 weeks following the estimated delivery date. Any premature termination of the pregnancy will be reported.

While pregnancy itself is not considered an AE or SAE, any pregnancy complication or elective termination of a pregnancy for medical reasons will be recorded as an AE or SAE.

A spontaneous abortion is always considered to be an SAE and will be reported as such. Furthermore, any SAE occurring as a result of a post-study pregnancy and is considered reasonably related to the study treatment by the investigator, will be reported to GSK as described in Section 13. While the investigator is not obligated to actively seek this information in former study participants, he or she may learn of an SAE through spontaneous reporting.

Any female subject who becomes pregnant while participating will be withdrawn from the study.

7.4.3. Action to be taken if pregnancy occurs in a female partner of a male study subject

The investigator will attempt to collect pregnancy information on any female partner of a male study subject who becomes pregnant while participating in this study. The investigator will record pregnancy information on the appropriate form and submit it to GSK within 2 weeks of learning of the partner's pregnancy. The partner will also be followed to determine the outcome of the pregnancy. Information on the status of the mother and child will be forwarded to GSK. Generally, follow-up will be no longer than 6 to 8 weeks following the estimated delivery date. Any premature termination of the pregnancy will be reported.

7.5. Pharmacokinetics

7.5.1. Blood Sample Collection

Blood samples for pharmacokinetic analysis of SB705498 will be collected at the time points indicated in Section 4.4, Time and Events Table. The actual date and time of each blood sample collection will be recorded.

Details of PK blood sample collection (including volume to be collected), processing, storage and shipping procedures are provided in the Study Procedures Manual (SPM).

7.5.2. Sample Analysis

Plasma analysis will be performed by Aptuit (Verona) srl, Italy. Concentrations of SB705498 will be determined in plasma using the currently approved analytical methodology. Raw data will be stored in the GLP Archives, Aptuit (Verona) srl.

8. LIFESTYLE AND/OR DIETARY RESTRICTIONS

8.1. Contraception Requirements

8.1.1. Female Subjects

Female subjects of childbearing potential must not become pregnant and so must be sexually inactive by abstinence or use contraceptive methods with a failure rate of < 1%.

Abstinence

Sexual inactivity by abstinence must be consistent with the preferred and usual lifestyle of the subject. Periodic abstinence (e.g. calendar, ovulation, symptothermal, post-ovulation methods) and withdrawal are not acceptable methods of contraception.

Contraceptive Methods with a Failure Rate of < 1%

- Oral contraceptive, either combined or progestogen alone
- Injectable progestogen
- Implants of levonorgestrel
- Estrogenic vaginal ring
- Percutaneous contraceptive patches
- Intrauterine device (IUD) or intrauterine system (IUS) that meets the <1% failure rate as stated in the product label
- Male partner sterilization (vasectomy with documentation of azoospermia) prior to the female subject's entry into the study, and this male is the sole partner for that subject. For this definition, “documented” refers to the outcome of the investigator's/designee’s medical examination of the subject or review of the subject's medical history for study eligibility, as obtained via a verbal interview with the subject or from the subject’s medical records.
- Double barrier method: condom and occlusive cap (diaphragm or cervical/vault caps) plus spermicidal agent (foam/gel/film/cream/suppository)

These allowed methods of contraception are only effective when used consistently, correctly and in accordance with the product label. The investigator is responsible for ensuring subjects understand how to properly use these methods of contraception.

8.1.2. Male Subjects

Male subjects with female partners of child-bearing potential must use one of the following contraceptive methods after the first dose of study treatment and until the end of the follow-up visit. :

- Condom plus partner use of a highly effective contraceptive such as occlusive cap (diaphragm or cervical/vault cap) plus spermicidal agent (foam/gel/film/cream/suppository), oral contraceptive, injectable progesterone, implant of etonogestrel or levonorgestrel, estrogenic vaginal ring, percutaneous contraceptive patches, or intrauterine device. **OR**
- Abstinence, defined as sexual inactivity consistent with the preferred and usual lifestyle of the subject. Periodic abstinence (e.g. calendar, ovulation, symptothermal, post-ovulation methods) and withdrawal are not acceptable methods of contraception

8.2. Meals and Dietary Restrictions

Subjects will be served a light breakfast, standard lunch and dinner in the Unit.

8.3. Caffeine, Alcohol, and Tobacco

- During each screening and dosing session, subjects will abstain from ingesting caffeine- or xanthine-containing products (e.g. coffee, tea, cola drinks and chocolate) for 24 hours prior to attending the unit until collection of the final pharmacokinetic and or pharmacodynamic sample during each session.
- During each screening and dosing session, subjects will abstain from alcohol for 24 hours prior to attending the unit until collection of the final pharmacokinetic and or pharmacodynamic sample during each session.
- Use of nicotine containing products is not allowed from 6 months prior to screening until after the final follow up visit.

8.4. Activity

Subjects will abstain from strenuous exercise for 48 hours prior to each blood collection for clinical laboratory tests (including screening). Subjects may participate in light recreational activities during studies (e.g., watch television, read).

Sun bed exposure and sunbathing must be avoided from 7 days prior to screening, the duration of the study and for a week after the last dose.

9. CONCOMITANT MEDICATIONS AND NON-DRUG THERAPIES

9.1. Permitted Medications

Paracetamol at doses of ≤ 2 grams/day is permitted. Other concomitant medication may be considered on a case by case basis by the GSK Medical Monitor.

9.2. Prohibited Medications

Subjects must abstain from taking prescription or non-prescription drugs (including vitamins, antihistamines and dietary or herbal supplements) within 7 days, (or 14 days if the drug is a potential enzyme inducer), or 5 half-lives (whichever is longer), prior to screening and the first dose of study medication and until completion of the follow-up visit, unless in the opinion of the Investigator and sponsor the medication will not interfere with the study.

Subjects should also avoid photosensitizing drugs (e.g, tetracycline, thiazides, fluoroquinolones, phenothiazines, or sulfaonamides).

9.3. Non-Drug Therapies

Subjects must abstain from taking any vitamins, herbal and dietary supplements within 7 days (or 14 days if the drug is a potential enzyme inducer) prior to screening or 5 half-lives (whichever is longer) prior to the first dose of study medication until completion of the follow-up visit, unless in the opinion of the Investigator and sponsor the medication will not interfere with the study.

10. COMPLETION OR EARLY WITHDRAWAL OF SUBJECTS

10.1. Subject Completion

A completed subject is one who has completed all phases of the study including the follow-up visit.

The end of the study is defined as the last subject's last visit.

10.2. Subject Withdrawal Criteria

Refer to Section 4.4 for dose adjustment/stopping criteria based on safety criteria.

A subject may withdraw from study treatment at any time at his/her own request, or may be withdrawn at any time at the discretion of the investigator for safety, behavioral or administrative reasons.

10.3. Subject Withdrawal Procedures

Subjects withdrawn from the study after receiving one or more doses of SB705498, will be asked to complete the safety assessments and the follow-up procedures as described in Section 4.3.

10.3.1. Subject Withdrawal from Study

Subjects will be withdrawn if they experience:

- An intercurrent illness that is considered to compromise either their safety and well-being or the scientific validity of the study Unacceptable AEs
- A change in status with regard to exclusion criteria
- A subject may voluntarily discontinue participation in the study at any time. The Investigator may also, at his or her discretion, discontinue the subject from participating in the study at any time.

Decision regarding replacement of subjects prematurely discontinued from study will be made by the Investigator and GSK Medical Monitor on a case-by-case basis.

10.3.2. Subject Withdrawal from Study Treatment

A subject will be considered to have prematurely withdrawn if he/she does not complete dosing for the entire planned treatment period for his group, having received the first dosing, for any reason. Once a subject has discontinued study drug, the subject may not re-enter the study.

10.4. Treatment After the End of the Study

Subjects will not receive any additional treatment from GSK after completion of the study because only healthy volunteers are eligible for study participation.

10.5. Screen and Baseline Failures

Data for screen and baseline failures will be collected in source documentation at the site.

11. STUDY TREATMENT

Study treatment dosage and administration details are listed in Section 4.

11.1. Blinding

This will be a double blind placebo study (for subject and Investigator team (CUC clinical staff) and un-blinded for the GSK study team and CUC pharmacy team not involved in clinical assessments. The investigator or treating physician may unblind a subject's treatment assignment only in the case of an emergency, when knowledge of the study treatment is essential for the appropriate clinical management or welfare of the subject. Whenever possible, the investigator must first discuss options with the GSK Medical Monitor or appropriate GSK study personnel before unblinding the subject's treatment assignment. If this is impractical, the investigator must notify GSK as soon as possible, but without revealing the treatment assignment of the unblinded subject, unless that information is important for the safety of subjects currently in the study. The date and reason for the unblinding must be recorded in the appropriate data collection tool.

GSK's Global Clinical Safety and Pharmacovigilance (GCSP) staff may unblind the treatment assignment for any subject with an SAE. If the SAE requires that an expedited regulatory report be sent to one or more regulatory agencies, a copy of the report, identifying the subject's treatment assignment, may be sent to clinical investigators in accordance with local regulations and/or GSK policy.

11.2. Packaging and Labeling

The contents of the label will be in accordance with all applicable regulatory requirements.

11.3. Preparation/Handling/Storage/Accountability

No special preparation of study treatment is required.

Study treatment must be dispensed or administered according to procedures described herein. Only subjects enrolled in the study may receive study treatment. Only authorised site staff may supply or administer study treatment. All study treatment must be stored in a secure area with access limited to the investigator and authorized site staff. Study treatment is to be stored between +2°C – +8°C (in amber glass bottles) whilst at the clinical site. The product can remain in amber glass bottles at controlled room temperature (20ºC - 25ºC, allowing excursions between 15ºC - 30ºC) for up to 48 hours prior to dosing. Maintenance of a temperature log (manual or automated) is required.

The investigator, institution, or the head of the medical institution (where applicable) is responsible for study treatment accountability, reconciliation, and record maintenance. The investigator or the head of the medical institution (where applicable), or designated site staff (e.g., storage manager, where applicable) must maintain study treatment accountability records throughout the course of the study. The responsible person(s) will document the amount of study treatment received from and returned to GSK and the amount supplied and/or administered to subjects. The required accountability unit for this study will be per bottle. Discrepancies are to be reconciled or resolved. Procedures for final disposition of unused study treatment are listed in the SPM.

Investigational product is not expected to pose significant occupational safety risk to site staff under normal conditions of use and administration. A Material Safety Data Sheet (MSDS)/equivalent document describing occupational hazards and recommended handling precautions either will be provided to the investigator, where this is required by local laws, or is available upon request from GSK.

However, precautions are to be taken to avoid direct skin contact, eye contact, and generating aerosols or mists. In the case of unintentional occupational exposure notify the monitor, medical monitor and/or study manager.

11.4. Assessment of Compliance

When the individual dose for a subject is prepared from a bulk supply, the preparation of the dose will be confirmed by a second member of the study site staff.

When subjects are dosed at the study site, they will receive study treatment directly from the investigator or designee, under medical supervision. The date and time of each dose administered in the clinic will be recorded in the source documents. The dose of study treatment and study participant identification will be confirmed at the time of dosing by a member of the study site staff other than the person administering the study treatment.

11.5. Treatment of Study Treatment Overdose

For this study, any dose of SB705498 > 250mg (10 applications of 5% cream) within a 24 hour time period [±1 hour] will be considered an overdose.

GSK does not recommend specific treatment for an overdose. The investigator will use clinical judgment to treat any overdose.

12. CHALLENGE AGENTS

In Part A Capsaicin will be used to induce flare and in Part B cowhage and histamine will be used to induce itch. A summary of the risk management for these challenge agents can be found in Section 1.7 and details on how this will be administered and handled are detailed in the Section 7.3

13. ADVERSE EVENTS (AE) AND SERIOUS ADVERSE EVENTS (SAE)

The investigator or site staff is responsible for detecting, documenting and reporting events that meet the definition of an AE or SAE.

AEs will be collected from the moment the informed consent form has been signed so that any issues relating to the challenge agent testing can be recorded and until the follow-up contact. Medical occurrences that begin prior to the start of study treatment but after obtaining informed consent may be recorded on the Medical History/Current Medical Conditions CRF.

SAEs will be collected over the same time period as stated above for AEs. However, any SAEs assessed as related to study participation (e.g. study treatment, protocol-mandated procedures, invasive tests, or change in existing therapy) or related to a GSK concomitant medication will be recorded from the time a subject consents to participate in the study up to and including any follow-up contact. All SAEs will be recorded and reported to GSK within 24 hours, as indicated in Section 13.7.

Investigators are not obligated to actively seek AEs or SAEs in former study participants. However, if the investigator learns of any SAE, including a death, at any time after a subject has been discharged from the study, and he/she considers the event reasonably related to the study treatment or study participation, the investigator would promptly notify GSK.

13.1. Definition of Adverse Events

An AE is any untoward medical occurrence in a patient or clinical investigation subject, temporally associated with the use of a medicinal product, whether or not considered related to the medicinal product.

Note: An AE can therefore be any unfavorable and unintended sign (including an abnormal laboratory finding), symptom, or disease (new or exacerbated) temporally associated with the use of a medicinal product.

Events meeting the definition of an AE **include**:

- Any abnormal laboratory test results (hematology, clinical chemistry, or urinalysis) or other safety assessments (e.g., ECGs, radiological scans, vital signs measurements), including those that worsen from baseline, and felt to be clinically significant in the medical and scientific judgement of the investigator.
- Exacerbation of a chronic or intermittent pre-existing condition including either an increase in frequency and/or intensity of the condition.
- New conditions detected or diagnosed after study treatment administration even though it may have been present prior to the start of the study.
- Signs, symptoms, or the clinical sequelae of a suspected interaction.

- Signs, symptoms, or the clinical sequelae of a suspected overdose of either study treatment or a concomitant medication (overdose per se will not be reported as an AE/SAE).
- "Lack of efficacy" or "failure of expected pharmacological action" per se will not be reported as an AE or SAE. However, the signs and symptoms and/or clinical sequelae resulting from lack of efficacy will be reported if they fulfil the definition of an AE or SAE.
- The signs and symptoms and/or clinical sequelae resulting from lack of efficacy will be reported if they fulfil the definition of an AE or SAE. In addition, "lack of efficacy" or "failure of expected pharmacological action" also constitutes an AE or SAE.

Events that **do not** meet the definition of an AE include:

- Any clinically significant abnormal laboratory findings or other abnormal safety assessments that are associated with the underlying disease, unless judged by the investigator to be more severe than expected for the subject's condition.
- The disease/disorder being studied or expected progression, signs, or symptoms of the disease/disorder being studied, unless more severe than expected for the subject's condition.
- Medical or surgical procedure (e.g., endoscopy, appendectomy); the condition that leads to the procedure is an AE.
- Situations where an untoward medical occurrence did not occur (social and/or convenience admission to a hospital).
- Anticipated day-to-day fluctuations of pre-existing disease(s) or condition(s) present or detected at the start of the study that do not worsen.

13.2. Definition of Serious Adverse Events

If an event is not an AE per Section 13.1, then it cannot be an SAE even if serious conditions are met (e.g., hospitalization for signs/symptoms of the disease under study, death due to progression of disease, etc).

An SAE is any untoward medical occurrence that, at any dose:

a. Results in death

b. Is life-threatening

   NOTE: The term 'life-threatening' in the definition of 'serious' refers to an event in which the subject was at risk of death at the time of the event. It does not refer to an event, which hypothetically might have caused death, if it were more severe.

c. Requires hospitalization or prolongation of existing hospitalization

   NOTE: In general, hospitalization signifies that the subject has been detained (usually involving at least an overnight stay) at the hospital or emergency ward for observation and/or treatment that would not have been appropriate in the physician's

office or out-patient setting. Complications that occur during hospitalization are AEs. If a complication prolongs hospitalization or fulfills any other serious criteria, the event is serious. When in doubt as to whether "hospitalization" occurred or was necessary, the AE should be considered serious.

Hospitalization for elective treatment of a pre-existing condition that did not worsen from baseline is not considered an AE.

d. Results in disability/incapacity, or

NOTE: The term disability means a substantial disruption of a person's ability to conduct normal life functions. This definition is not intended to include experiences of relatively minor medical significance such as uncomplicated headache, nausea, vomiting, diarrhea, influenza, and accidental trauma (e.g. sprained ankle) which may interfere or prevent everyday life functions but do not constitute a substantial disruption.

e. Is a congenital anomaly/birth defect

f. Medical or scientific judgment should be exercised in deciding whether reporting is appropriate in other situations, such as important medical events that may not be immediately life threatening or result in death or hospitalization but may jeopardize the subject or may require medical or surgical intervention to prevent one of the other outcomes listed in the above definition. These should also be considered serious. Examples of such events are invasive or malignant cancers, intensive treatment in an emergency room or at home for allergic bronchospasm, blood dyscrasias or convulsions that do not result in hospitalization, or development of drug dependency or drug abuse.

g. Is associated with liver injury **and** impaired liver function defined as:

- ALT ≥ 3xULN, and
- Total bilirubin ≥ 2xULN or INR > 1.5.

NOTES:

Bilirubin fractionation should be performed if testing is available. If fractionation is unavailable, urinary bilirubin is to be measured via dipstick (a measurement of direct bilirubin, which would suggest liver injury).

INR measurement is not required; if measured, the threshold value stated will not apply to patients receiving anticoagulants. If INR measurement is obtained, the value is to be recorded on the SAE form.

- Refer to Section 14 for the required liver chemistry follow-up instructions.

13.3. Method of Detecting AEs and SAEs

Care will be taken not to introduce bias when detecting AEs and/or SAEs. Open-ended and non-leading verbal questioning of the subject is the preferred method to inquire about AE occurrence. Appropriate questions include:

- "How are you feeling?"
- "Have you had any (other) medical problems since your last visit/contact?"

- “Have you taken any new medicines, other than those provided in this study, since your last visit/contact?”

13.4. Recording of AEs and SAEs

When an AE/SAE occurs, it is the responsibility of the investigator to review all documentation (e.g., hospital progress notes, laboratory, and diagnostics reports) relative to the event. The investigator will then record all relevant information regarding an AE/SAE in the appropriate data collection tool.

It is not acceptable for the investigator to send photocopies of the subject’s medical records to GSK in lieu of completion of the GSK, AE/SAE data collection tool. However, there may be instances when copies of medical records for certain cases are requested by GSK. In this instance, all subject identifiers, with the exception of the subject number, will be blinded on the copies of the medical records prior to submission of to GSK.

The investigator will attempt to establish a diagnosis of the event based on signs, symptoms, and/or other clinical information. In such cases, the diagnosis will be documented as the AE/SAE and not the individual signs/symptoms.

Subject-completed health outcomes questionnaires and the collection of AE data are independent components of the study. Responses to each question in the health outcomes questionnaire will be treated in accordance with standard scoring and statistical procedures detailed by the scale’s developer. The use of a single question from a multidimensional health survey to designate a cause-effect relationship to an AE is inappropriate.

13.5. Evaluating AEs and SAEs

13.5.1. Assessment of Intensity

The investigator will make an assessment of intensity for each AE and SAE reported during the study and will assign it to one of the following categories:

Mild: An event that is easily tolerated by the subject, causing minimal discomfort and not interfering with everyday activities.

Moderate: An event that is sufficiently discomforting to interfere with normal everyday activities.

Severe: An event that prevents normal everyday activities.

An AE that is assessed as severe will not be confused with an SAE. Severity is a category utilized for rating the intensity of an event; and both AEs and SAEs can be assessed as severe. An event is defined as ‘serious’ when it meets at least one of the pre-defined outcomes as described in the definition of an SAE.

13.5.2. Assessment of Causality

The investigator is obligated to assess the relationship between study treatment and the occurrence of each AE/SAE. A "reasonable possibility" is meant to convey that there are facts/evidence or arguments to suggest a causal relationship, rather than a relationship cannot be ruled out. The investigator will use clinical judgment to determine the relationship. Alternative causes, such as natural history of the underlying diseases, concomitant therapy, other risk factors, and the temporal relationship of the event to the study treatment will be considered and investigated. The investigator will also consult the Investigator Brochure (IB) and/or Product Information, for marketed products, in the determination of his/her assessment.

There may be situations when an SAE has occurred and the investigator has minimal information to include in the initial report to GSK. However, **it is very important that the investigator always make an assessment of causality for every event prior to the initial transmission of the SAE data to GSK.** The investigator may change his/her opinion of causality in light of follow-up information, amending the SAE data collection tool accordingly. The causality assessment is one of the criteria used when determining regulatory reporting requirements.

13.6. Follow-up of AEs and SAEs

After the initial AE/SAE report, the investigator is required to proactively follow each subject at subsequent visits/contacts. All AEs and SAEs will be followed until resolution, until the condition stabilizes, until the event is otherwise explained, or until the subject is lost to follow-up.

The investigator is obligated to perform or arrange for the conduct of supplemental measurements and/or evaluations as may be indicated or as requested by GSK to elucidate as fully as possible the nature and/or causality of the AE or SAE. The investigator is obligated to assist. This may include additional laboratory tests or investigations, histopathological examinations or consultation with other health care professionals. If a subject dies during participation in the study or during a recognized follow-up period, the investigator will provide GSK with a copy of any post-mortem findings, including histopathology.

New or updated information will be recorded in the originally completed data collection tool. The investigator will submit any updated SAE data to GSK within the designated reporting periods.

13.7. Prompt Reporting of SAEs to GSK

Once the investigator determines that an event meets the protocol definition of an SAE, the SAE will be reported to GSK **within 24 hours**. Any follow-up information on a previously reported SAE will also be reported to GSK within 24 hours.

If the investigator does not have all information regarding an SAE, he/she will not wait to receive additional information before notifying GSK of the event and completing the appropriate data collection tool. The investigator will always provide an assessment of causality at the time of the initial report as described in Section 13.5.2, Assessment of Causality.

Facsimile transmission of the following PIMS listings for the corresponding subject is the preferred method to transmit SAE information to the GSK Medical monitor or protocol contact:

- SAE listing
- Demographic listing
- Investigational product listing

In rare circumstances and in the absence of facsimile equipment, notification by telephone is acceptable, with a copy of all required information sent by overnight mail. Initial notification via the telephone does not replace the need for the investigator to complete and sign the SAE data collection tool within the designated reporting periods.

If the PIMS system is unavailable when the SAE occurs, the site will use the paper SAE form and fax that to the GSK medical monitor or protocol contact. The site will enter the SAE data into PIMS as soon as the system becomes available.

GSK contacts for SAE receipt can be found at this beginning of this protocol on the Sponsor/Medical Monitor Contact Information page.

13.8. Regulatory Reporting Requirements for SAEs

Prompt notification of SAEs by the investigator to GSK is essential so that legal obligations and ethical responsibilities towards the safety of subjects are met.

GSK has a legal responsibility to notify both the local regulatory authority and other regulatory agencies about the safety of a product under clinical investigation. GSK will comply with country specific regulatory requirements relating to safety reporting to regulatory authorities, IRBs/IECs and investigators.

Investigator safety reports are prepared for suspected unexpected serious adverse reactions according to local regulatory requirements and GSK policy and are forwarded to investigators as necessary. An investigator who receives an investigator safety report describing an SAE(s) or other specific safety information (e.g., summary or listing of SAEs) from GSK will file it with the IB and will notify the IRB/IEC, if appropriate according to local requirements.

14. LIVER CHEMISTRY FOLLOW-UP PROCEDURES

Refer to the diagram in Appendix 1 for a visual presentation of the procedures listed below.

The procedures listed below are to be followed if a subject meets the liver chemistry stopping criteria defined in Section 4.4.1.1:

- Immediately and permanently withdraw the subject from study treatment
- Notify the GSK medical monitor within 24 hours of learning of the abnormality to confirm the subject's study treatment cessation and follow-up.

- Complete the "Safety Follow-Up Procedures" listed below.
- Complete the liver event case report forms. If the event also meets the criteria of an SAE (see Section 13.2), the SAE data collection tool will be completed separately with the relevant details.
- Upon completion of the safety follow-up permanently withdraw the subject from the study and do not rechallenge with study treatment.

Safety Follow-Up Procedures for subjects with ALT ≥ 3xULN:

- Monitor subjects <u>weekly</u> until liver chemistries (ALT, AST, alkaline phosphatase, bilirubin) resolve, stabilize or return to within baseline values.

Safety Follow-Up Procedures for subjects with ALT ≥3xULN and bilirubin ≥2xULN (or ALT ≥ 3xULN and INR[1] **> 1.5):**

- <u>This event is considered an SAE</u> (see Section 13.2). Serum bilirubin fractionation should be performed if testing is available. If fractionation is unavailable, urinary bilirubin is to be measured via dipstick (a measurement of direct bilirubin, which would suggest liver injury).
- Make every reasonable attempt to have subjects return to the clinic within 24 hours for repeat liver chemistries, additional testing, and close monitoring (with specialist or hepatology consultation recommended).
- Monitor subjects <u>twice weekly</u> until liver chemistries (ALT, AST, alkaline phosphatase, bilirubin) resolve, stabilize or return to within baseline values.

In addition, for <u>all</u> subjects with ALT ≥ 3xULN, every attempt must be made to also obtain the following:

- Viral hepatitis serology including:
  - Hepatitis A IgM antibody.
  - Hepatitis B surface antigen and Hepatitis B Core Antibody (IgM).
  - Hepatitis C RNA.
  - Cytomegalovirus IgM antibody.
  - Epstein-Barr viral capsid antigen IgM antibody (or if unavailable, obtain heterophile antibody or monospot testing).
  - Hepatitis E IgM antibody.
- Blood sample for pharmacokinetic (PK) analysis, obtained within 24hrs of last dose. Record the date/time of the PK blood sample draw and the date/time of the last dose of study treatment prior to blood sample draw on the CRF. If the date or time of the last dose is unclear, provide the subject's best approximation. If the date/time of the

[1] INR testing not required per protocol and the threshold value does not apply to subjects receiving anticoagulants.

last dose cannot be approximated <u>OR</u> a PK sample cannot be collected in the time period indicated above, **do not obtain a PK sample**. Instructions for sample handling and shipping are included in the SPM.

- Serum creatine phosphokinase (CPK) and lactate dehydrogenase (LDH).
- Fractionate bilirubin, if total bilirubin ≥ 2xULN.
- Assess eosinophilia
- Record the appearance or worsening of clinical symptoms of hepatitis (fatigue, nausea, vomiting, right upper quadrant pain or tenderness, fever, rash or eosinophilia) on the AE CRF.
- Record use of concomitant medications, acetaminophen, herbal remedies, other over the counter medications, or putative hepatotoxins on the Concomitant Medications CRF.
- Record alcohol use on the Liver Events CRF.

The following are required for subjects with ALT ≥ 3xULN **and** bilirubin ≥ 2xULN but are optional for other abnormal liver chemistries:

- Anti-nuclear antibody, anti-smooth muscle antibody, and Type 1 anti-liver kidney microsomal antibodies.
- Liver imaging (ultrasound, magnetic resonance, or computerized tomography) to evaluate liver disease.
- The Liver Imaging and/or Liver Biopsy CRFs are also to be completed if these tests are performed.

15. STUDY CONDUCT CONSIDERATIONS

15.1. Posting of Information on Clinicaltrials.gov

Study information from this protocol will be posted on clinicaltrials.gov before enrollment of subjects begins.

15.2. Regulatory and Ethical Considerations, Including the Informed Consent Process

GSK will obtain favorable opinion/approval to conduct the study from the appropriate regulatory agency in accordance with any applicable country-specific regulatory requirements prior to a site initiating the study in that country.

The study will be conducted in accordance with all applicable regulatory requirements.

The study will also be conducted in accordance with ICH Good Clinical Practice (GCP), all applicable subject privacy requirements, and, the guiding principles of the 2008 Declaration of Helsinki. This includes, but is not limited to, the following:

- IRB/IEC review and favorable opinion/approval to conduct the study and of any subsequent relevant amended documents
- Written informed consent (and any amendments) to be obtained for each subject before participation in the study
- Investigator reporting requirements (e.g. reporting of AEs/SAEs/protocol deviations to IRB/IEC)

15.2.1. Urgent Safety Measures

If an event occurs that is related to the conduct of the study or the development of the study treatment, and this new event is likely to affect the safety of subjects, the sponsor and the investigator will take appropriate urgent safety measures to protect subjects against any immediate hazard.

The sponsor will work with the investigator to ensure the IEC/IRB is notified.

15.3. Quality Control (Study Monitoring)

In accordance with applicable regulations including GCP, and GSK procedures, GSK monitors will contact the site prior to the start of the study to review with the site staff the protocol, study requirements, and their responsibilities to satisfy regulatory, ethical, and GSK requirements. When reviewing data collection procedures, the discussion will also include identification, agreement and documentation of data items for which the CRF will serve as the source document.

GSK will monitor the study and site activity to verify that the:

- Data are authentic, accurate, and complete.
- Safety and rights of subjects are being protected.
- Study is conducted in accordance with the currently approved protocol and any other study agreements, GCP, and all applicable regulatory requirements.

The investigator and the head of the medical institution (where applicable) agrees to allow the monitor direct access to all relevant documents

15.4. Quality Assurance

To ensure compliance with GCP and all applicable regulatory requirements, GSK may conduct a quality assurance audit. Regulatory agencies may also conduct a regulatory inspection of this study. Such audits/inspections can occur at any time during or after completion of the study. If an audit or inspection occurs, the investigator and institution agree to allow the auditor/inspector direct access to all relevant documents and to allocate his/her time and the time of his/her staff to the auditor/inspector to discuss findings and any relevant issues.

15.5. Study and Site Closure

Upon completion or premature discontinuation of the study, the monitor will conduct site closure activities with the investigator or site staff, as appropriate, in accordance with applicable regulations including GCP, and GSK procedures.

In addition, GSK reserves the right to temporarily suspend or prematurely discontinue this study at any time for reasons including, but not limited to, safety or ethical issues or severe non-compliance. If GSK determines such action is needed, GSK will discuss this with the investigator or the head of the medical institution (where applicable), including the reasons for taking such action. When feasible, GSK will provide advance notification to the investigator or the head of the medical institution, where applicable, of the impending action prior to it taking effect.

If the study is suspended or prematurely discontinued for safety reasons, GSK will promptly inform investigators or the head of the medical institution (where applicable) and the regulatory authorities of the suspension or premature discontinuation of the study and the reason(s) for the action. If required by applicable regulations, the investigator or the head of the medical institution (where applicable) must inform the IRB/IEC promptly and provide the reason for the suspension or premature discontinuation.

15.6. Records Retention

Following closure of the study, the investigator or the head of the medical institution (where applicable) must maintain all site study records, except for those required by local regulations to be maintained by someone else, in a safe and secure location. The records must be maintained to allow easy and timely retrieval, when needed (e.g., audit or inspection), and, whenever feasible, to allow any subsequent review of data in conjunction with assessment of the facility, supporting systems, and staff. Where permitted by local laws/regulations or institutional policy, some or all of these records can be maintained in a format other than hard copy (e.g., microfiche, scanned, electronic); however, caution needs to be exercised before such action is taken. The investigator must assure that all reproductions are legible and are a true and accurate copy of the original and meet accessibility and retrieval standards, including re-generating a hard copy, if required. Furthermore, the investigator must ensure there is an acceptable back up of these reproductions and that an acceptable quality control process exists for making these reproductions.

GSK will inform the investigator of the time period for retaining these records to comply with all applicable regulatory requirements. The minimum retention time will meet the strictest standard applicable to that site for the study, as dictated by any institutional requirements or local laws or regulations, or GSK standards/procedures; otherwise, the retention period will default to 15 years.

The investigator must notify GSK of any changes in the archival arrangements, including, but not limited to, archival at an off-site facility or transfer of ownership of the records in the event the investigator leaves the site.

15.7. Provision of Study Results to Investigators, Posting to the Clinical Trials Register and Publication

Where required by applicable regulatory requirements, an investigator signatory will be identified for the approval of the clinical study report. The investigator will be provided reasonable access to statistical tables, figures, and relevant reports and will have the opportunity to review the complete study results at a GSK site or other mutually agreeable location.

GSK will also provide the investigator with the full summary of the study results. The investigator is encouraged to share the summary results with the study subjects, as appropriate.

GSK will provide the investigator with the randomization codes for their site only after completion of the full statistical analysis.

The results summary will be posted to the Clinical Study Register at the time of the first regulatory approval or within 12 months of any decision to terminate development. In addition, a manuscript will be submitted to a peer-reviewed journal for publication no later than 12 months after the first approval or any decision to terminate development. When manuscript publication in a peer-reviewed journal is not feasible, further study information will be posted to the GSK Clinical Study Register to supplement the results summary.

15.8. Data Management

GSK Data Management will identify and implement the most effective data acquisition and management strategy for each clinical trial protocol and deliver datasets which support the protocol objectives. Subject data will be entered into GSK defined CRFs and combined with data provided from other sources (e.g. diary data, laboratory data) in a validated data system. Subject initials will not be transmitted to GSK for inclusion in the datasets. Clinical data management will be performed in accordance with applicable GSK standards and data cleaning procedures with the objective of removing errors and inconsistencies in the data which would otherwise affect the analysis and reporting objectives, or the credibility of the Clinical Study Report. Adverse events and concomitant medications terms will be coded using validated dictionaries. Original CRFs will be retained by GSK, while the investigator will retain a copy.

16. REFERENCES

A.D.P. Papoiu, H.L. Tey, R.C. Coghill, H. Wang, G. Yosipovitch Cowhage-induced itch as an experimental model for pruritus. A comparative study with histamine-induced itch PLoS One, 6 (3) (2011), p. e17786

Anand P., Bley K.(2011) Topical capsaicin for pain management: Therapeutic potential and mechanisms of action of the new high-concentration capsaicin 8 patch British Journal of Anaesthesia 107:4 (490-502)

Bíró T, Tóth BI, Marincsák R, Dobrosi,N Géczy T, Paus R (2007)TRP channels as novel players in the pathogenesis and therapy of itch Biochim. Biophys. Acta - Molecular Basis of Disease, 1772:1004-1021

Bley KR. (2010) TRPV1 agonist approaches for pain management. In: Gomtsyan A, Faltynek CR, editors. Vanilloid Receptor TRPV1 in Drug Discovery: Targeting Pain and Other Pathological Disorders. New York: Wiley;. p. 325-47.

Bode A M, Cho Y Y, Zheng D. Transient Receptor Potential Type Vanilloid 1 Suppresses Skin Carcinogenesis. *Cancer Res.* 2009;69:905-913

Chizh B.A., O'Donnell M.B., Napolitano A., Wang J., Brooke A.C., Aylott M.C., Bullman J.N., Gray E.J., Lai R.Y., Williams P.M., Appleby J.M. The effects of the TRPV1 antagonist on TRPV1 receptor-mediated activity and inflammatory hyperalgesia in humans Pain 2007 132: 132-141

Drake LA, Fallon JD, Sober A. Relief of pruritus in patients with atopic dermatitis after treatment with topical doxn cream. The Doxepin Study Group. J Am Acad Dermatol 1994;31:613-16; .

Drake LA, Millikan LE. The antipruritic effect of 5% doxepin cream in patients with eczematous dermatitis. Doxepin Study Group. Arch Dermatol 1995;131:140)

GlaxoSmithKline Document Number GH2007/00009/00 Study ID VR1111611. A randomised, double-blind, placebo-controlled study to assess the effect of oral, single dose SB-705498 in a validated intranasal capsaicin challenge model in healthy volunteers. Report Date 01-APR-2008.

GlaxoSmithKline Document Number HM2005/00272/00 Study ID SB-705498/001. A randomised, single-blind, placebo-controlled, dose escalation study to investigate the safety, tolerability and pharmacokinetics of single doses of SB-705498, a novel vanilloid receptor antagonist in healthy volunteers. Report Date 07-Jul-2006.

GlaxoSmithKline Document Number HM2004/00078/07 Study ID SB705498. Investigator Brochure. Report Date 02 Aug 2010.

Hutter MM, Wick EC, Day AL et al. (2005) Transient receptor potential vanilloid (TRPV-1) promotes neurogenic inflammation in the pancreas via activation of the neurokinin-1 receptor (NK-1R). Pancreas 30:260–265

Imamachi N, Park GH, Lee H et al. (2009) TRPV1-expressing primary afferents generate behavioral responses to pruritogens via multiple mechanisms. Proc Natl Acad Sci USA 106:11330–11335

Ishiuji Y, Coghill RC, Patel TS, Oshiro Y, Kraft RA, et al. (2009) Distinct patterns of brain activity evoked by histamine-induced itch reveal an association with itch intensity).

Kawana S, Yamaoka J. Rapid changes in substance P signaling and neutral endopeptidase induced by skin-scratching stimulation in mice. *Dermatol Sci.* 2007;48:123-132.

Kofler L, Ulmer H, Kofler H. 50-Skin-Prick Test: A Tool to Diagnose Histamine Intolerance. *Scholarly Research Network ISRN Allergy.* 2011;2011:353045.

Li Y J, Peng j, Luo X J. Recent advances in the study on capsaicinoids and capsinoids. *European Journal of Pharmacology.* 2011;650:1-7

Lukas Kofler, Hanno Ulmer, and Heinz Kofler, "Histamine 50-Skin-Prick Test: A Tool to Diagnose Histamine Intolerance," *ISRN Allergy*, vol. 2011, Article ID 353045, 5 pages, 2011. doi:10.5402/2011/353045

M. Steinhoff, U. Neisius, A. Ikoma, M. Fartasch, G. Heyer, P.S. Skov, T.A. Luger, M. Schmelz Proteinase-activated receptor-2 mediates itch: a novel pathway for pruritus in human skin J. Neurosci., 23 (2003), pp. 6176–6180

Melton FM, Shelley WB (1950) The effect of topical antipruritic therapy on experimentally induced pruritus in man. J Invest Dermatol 15: 325–332

Namer B, Carr R, Johanek LM (2008) Separate peripheral pathways for pruritus in man. J Neurophysiol 100: 2062–2069.;

Nolano M, Simone DA, Wendelschafer-Crabb G, Johnson T, Hazen E, Kennedy WR: (1999) Topical capsaicin in humans: Parallel loss of epidermal nerve fibers and pain sensation. Pain 81:135-145,

Ohmura T, Hayashi T, Satoh Y et al. (2004) Involvement of substance P in scratching behaviour in an atopic dermatitis model. Eur J Pharmacol 491:191–194

Patel & Yosipovitch, Tran, W, Papoiu, A.D.P, Russoniello,C.V, Wang, H, Chan, Y.H,. Effect of itch, scratching and mental stress on autonomic nervous system function in atopic dermatitis. *Acta Dermato-Venereologica.* 2011;90:354.

P. Sikand, S.G. Shimada, B.G. Green, R.H. LaMotte Similar itch and nociceptive sensations evoked by punctate cutaneous application of capsaicin, histamine and cowhage Pain, 144 (2009), pp. 66–75

R.H. LaMotte, S.G. Shimada, B.G. Green, D. Zelterman Pruritic and nociceptive sensations and dysesthesias from a spicule of cowhage J. Neurophysiol., 101 (2009), pp. 1430–1443

Reddy et al., J. Neuroscience (2008b) 28:4331-5.)

Reddy V.B, Iuga A.O, Shimada S.G, LaMotte R.H, Lerner E.A. Cowhage-evoked itch is mediated by a novel cysteine protease: a ligand of protease-activated receptors J. Neurosci., 28 (2008a), pp. 4331–4335

Schmelz M. Itch and pain (2010) Neurosci Biobehav Rev 34: 171–176.)

Shim WS, Tak MH, Lee MH et al. (2007) TRPV1 mediates histamine-induced itching via the activation of phospholipase A2 and 12-lipoxygenase. J Neurosci 27:2331–2337

Steinhoff M, Bienenstock J, Schmelz M et al. (2006) Neurophysiological, neuroimmunological, and neuroendocrine basis of pruritus. J Invest Dermatol 126:1705–1718

Tejesh Patel & Gil Yosipovitch Expert Opin. Pharmacother. (2010) 11(10):1673-1682

Velazquez RA, McCarson KE, Cai Y et al. (2002) Upregulation of neurokinin-1 receptor expression in rat spinal cord by an N-terminal metabolite of substance P. Eur J Neurosci 16:229–241 |

Wang H, Papoiu ADP, Coghill RC, Patel T, Wang N and Yosipovitch G.(2010) British Journal Dermatology Ethnic differences in pain, itch and thermal detection in response to topical capsaicin: African Americans display a notably limited hyperalgesia and neurogenic inflammation 162 (5) 1023–1029,

Yamaoka J, Kawana S (2007) Rapid changes in substance P signaling and neutral endopeptidase induced by skin-scratching stimulation in mice. J Dermatol Sci 48:123–132

Yosipovitch G, Goon A, Wee J, The prevalence and clinical characteristics of pruritus among patients with extensive psoriasis. Br J Dermatol 2000;143:969-73

Yun J-W, Seo JA, Jang W-H, KohHJ, Bae I-H, Park YH, LimK-M Antipruritic Effects of TRPV1 Antagonist in Murine Atopic Dermatitis and Itching Models Journal of Investigative Dermatology (2011a) 131, 1576–1579;; published online 7 April 2011

Yun J-W, Seo JA, Jeong YS et al. (2011b) TRPV1 antagonist can suppress the atopic dermatitis-like symptoms by accelerating skin barrier recovery. J Dermatol Sci 62:8–15

APPENDICES

Appendix 1: Liver Safety Algorithms

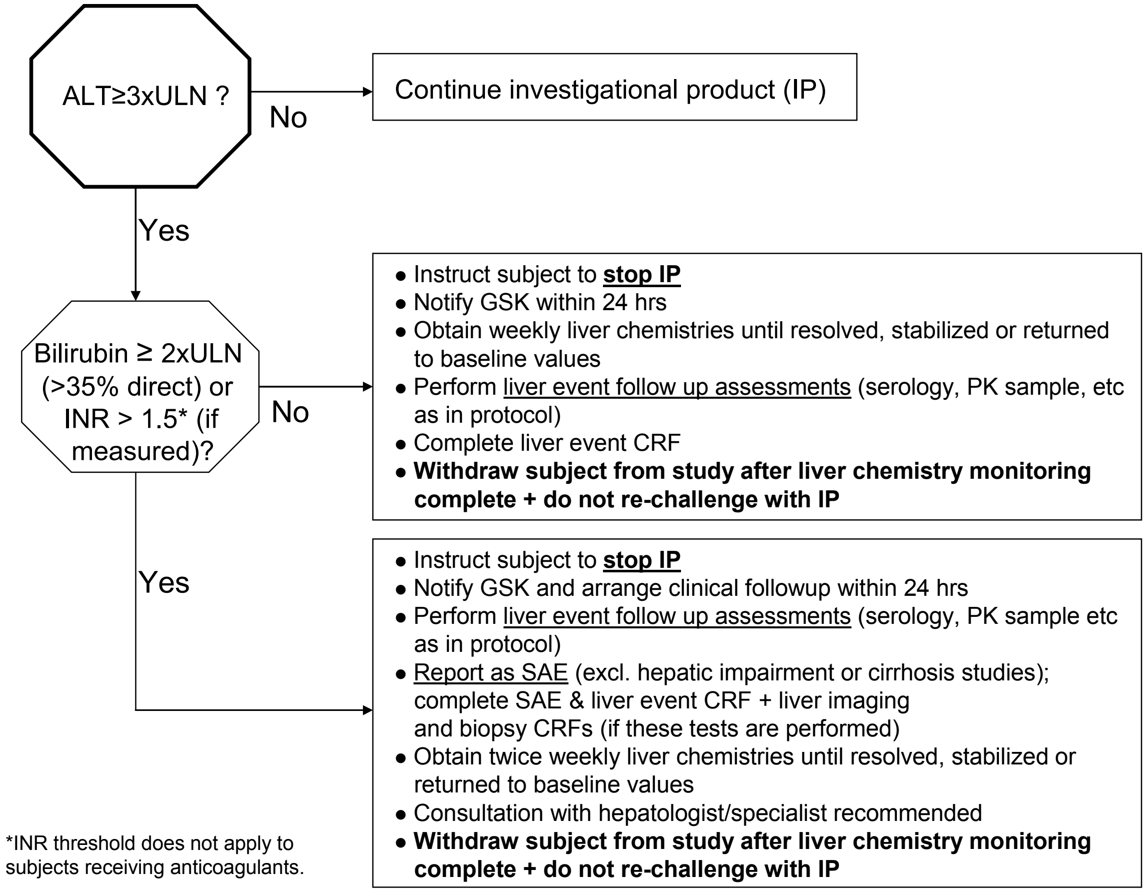

Appendix 2: Protocol Amendment Changes

Amendment 1

Where the Amendment Applies

Section 4.4.1.2 QTc withdrawal criteria and section 5.2.1 Inclusion Criteria.

List of Specific Changes

Section 4.4.1.2 QTc Withdrawal Criteria

PREVIOUS TEXT

- QTcB 500 msec or uncorrected QT >600 msec
- If subject has underlying bundle branch block then the QTc withdrawal criteria depends on the baseline value:

Baseline QTc value (with underlying bundle branch block)	QTc withdrawal criteria
<450 ms	>500 ms
450-480 ms	>530 ms

Withdrawal decisions are to be based on an average QTc value of triplicate ECGs. If an ECG demonstrates a prolonged QT interval, obtain 2 more ECGs over a brief period, and then use the averaged QTc values of the 3 ECGs to determine whether the subject should be discontinued from the study. Subjects who are withdrawn, will not return for a second session.

REVISED TEXT

- QTc, QTcB, QTcF > 500 msec or uncorrected QT>600msec
- Prolongation of QTcB or QTcF by > 60 msec as compared to baseline
- If subject has underlying bundle branch block then the QTc withdrawal criteria depends on the baseline value:

Baseline QTc value (with underlying bundle branch block)	QTc withdrawal criteria
<450 ms	>500 ms

Withdrawal decisions are to be based on an average QTc value of triplicate ECGs. If an ECG demonstrates a prolonged QT interval, obtain 2 more ECGs over a brief period, and then use the averaged QTc values of the 3 ECGs to determine whether the subject should be discontinued from the study. Subjects who are withdrawn, will not return for a second session.
